# Rapid RNA–ligand interaction analysis through high-information content conformational and stability landscapes

**DOI:** 10.1038/ncomms9898

**Published:** 2015-12-07

**Authors:** Nathan J. Baird, James Inglese, Adrian R. Ferré-D'Amaré

**Affiliations:** 1 National Heart, Lung and Blood Institute, NIH, 50 South Dr, Bethesda, Maryland 20892, USA; 2 National Center for Advancing Translational Sciences, NIH, 9800 Medical Center Dr, Rockville, Maryland, 20850, USA

## Abstract

The structure and biological properties of RNAs are a function of changing cellular conditions, but comprehensive, simultaneous investigation of the effect of multiple interacting environmental variables is not easily achieved. We have developed an efficient, high-throughput method to characterize RNA structure and thermodynamic stability as a function of multiplexed solution conditions using Förster resonance energy transfer (FRET). In a single FRET experiment using conventional quantitative PCR instrumentation, 19,400 conditions of MgCl_2_, ligand and temperature are analysed to generate detailed empirical conformational and stability landscapes of the cyclic diguanylate (c-di-GMP) riboswitch. The method allows rapid comparison of RNA structure modulation by cognate and non-cognate ligands. Landscape analysis reveals that kanamycin B stabilizes a non-native, idiosyncratic conformation of the riboswitch that inhibits c-di-GMP binding. This demonstrates that allosteric control of folding, rather than direct competition with cognate effectors, is a viable approach for pharmacologically targeting riboswitches and other structured RNA molecules.

## Abstract (web summary)

The structure and biological properties of RNAs are a function of changing cellular conditions. Here, Baird *et al.* report a high-throughput Förster resonance energy transfer (FRET) method to rapidly compare RNA structure modulation by cognate and non-cognate ligands across multiplexed solution conditions.

## Graphical abstract

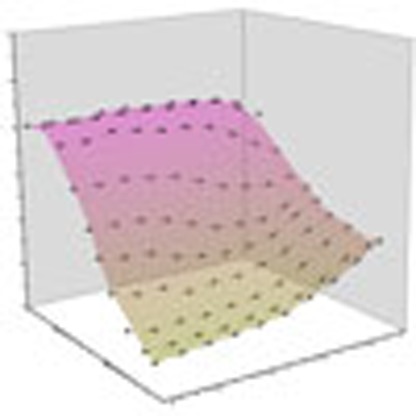

Many cellular RNAs adopt distinct global and local conformations in response to environmental variables such as temperature, ionic strength, the identity and concentrations of divalent cations and pH, as well as their specific or nonspecific binding to small molecules, proteins and other RNAs^1^,^2^,^3^,^4^,^5^,^6^,^7^,^8^. Indeed, the non-coding gene-regulatory mRNA elements known as riboswitches function by adopting different three-dimensional (3D) structures in the absence and presence of their cognate ligands^9^. Although non-coding RNAs, in general, and riboswitches, in particular, constitute attractive new targets for the development of drugs^10^,^11^,^12^,^13^,^14^, characterization of their complex responses to their environment is challenging. One-dimensional experiments that read out the response to one variable at a time may fail to uncover important facets of the molecular behaviour of an RNA, and even sparse sampling of its higher-dimensional conformational (and underlying free energy) landscape can be tedious and material intensive.

We have developed an efficient method to perform multi-dimensional biochemical and biophysical characterization of RNAs in a single high-throughput experiment. Our approach is based on Förster resonance energy transfer (FRET) and can be implemented on a conventional quantitative PCR (qPCR) instrument. We demonstrate that a single experiment probing 19,400 multiplexed solution conditions yields high-information content 3D and four-dimensional (4D) landscapes describing RNA conformation and tertiary structure stability as a function of Mg^2+^ concentration and ligand binding. We employ this approach to elucidate how the conformation and stability landscapes of a cyclic diguanylate (c-di-GMP) riboswitch are modulated by cognate and non-cognate ligands. In addition to delineating the solution parameter range over which the RNA exhibits maximal response, our multi-dimensional analysis uncovered an alternative, inhibitory riboswitch conformation stabilized by the aminoglycoside kanamycin B, a non-cognate ligand. Additional biophysical experiments demonstrated that the non-native aminoglycoside-induced conformation modulates binding of the cognate ligand to the riboswitch. Our method for rapidly determining conformational and stability landscapes of RNA thus suggests an approach to control riboswitch function through the stabilization of off-pathway conformations using non-cognate ligands. Our method should be broadly and generally applicable for identifying small-molecule ligands exerting conformational control of functional RNAs.

## Results

### Complex conformational switching of a c-di-GMP-I riboswitch

**Figure 1:**
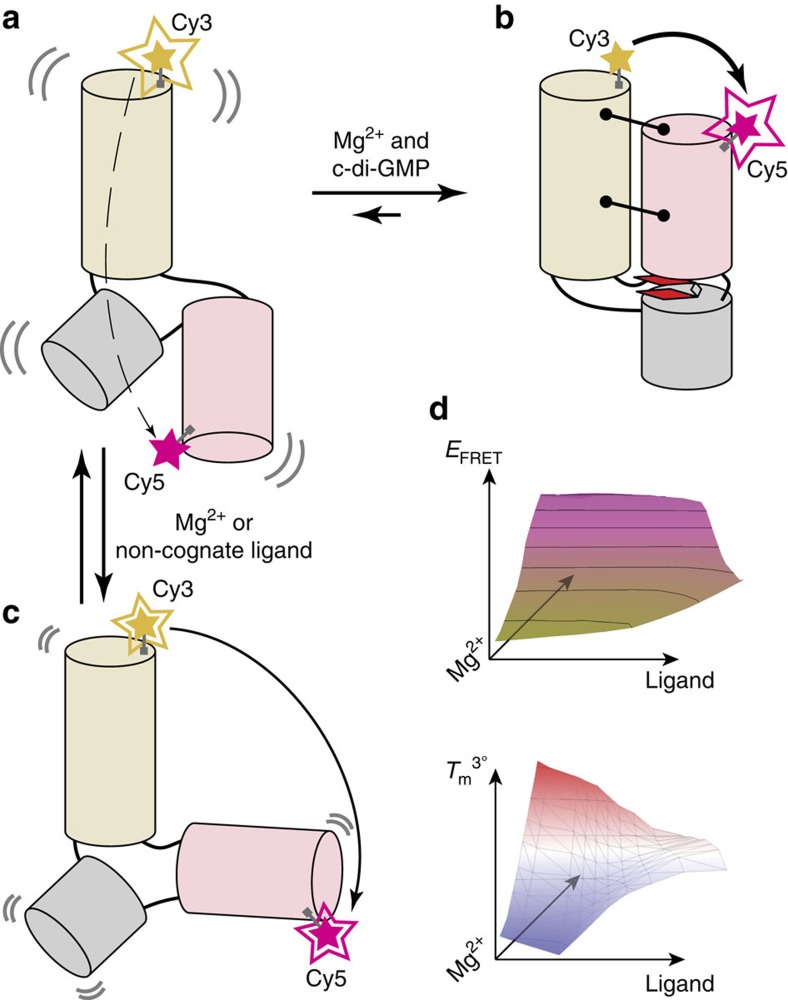
The c-di-GMP-I riboswitch aptamer domain undergoes a large conformational change in response to ligand binding. Schematics of the free (**a**) and ligand-bound (**b**) conformations of the RNA based on previous crystallographic, small-angle X-ray scattering (SAXS), single-molecule FRET (smFRET) and enzymatic cleavage experiments^16^,^19^. Alternative conformations (**c**) may be stabilized under certain conditions (for example, non-cognate ligand binding). Fluorophores (Cy3, yellow and Cy5, magenta) were covalently attached to the RNA in locations that would yield large FRET changes upon folding induced by changes in c-di-GMP or Mg^2+^ concentrations. (**a**) In the absence of c-di-GMP or Mg^2+^, Cy3 emission is much stronger than Cy5 emission. (**b**) Folding to the c-di-GMP-bound conformation (tertiary contacts, black lines terminating with circles; cyclic di-GMP, red, bound between two coaxially stacked helices) results in inversion of Cy3 and Cy5 fluorescence intensities, due to FRET. (**c**) Other conformations may yield intermediate fluorescence intensities. (**d**) RNA–ligand interactions can be characterized through multi-dimensional analysis of RNA conformations (FRET efficiency, *E*_FRET_) and thermal stability of the tertiary structure (*T*_m_^3°^).

The class I c-di-GMP (c-di-GMP-I) riboswitch^15^ regulates genes involved in motility, virulence and biofilm formation in pathogenic bacteria in response to intracellular levels of the second messenger c-di-GMP. Ligand binding-induced conformation transitions of the aptamer domain of this riboswitch have previously been described^16^,^17^. Enzymatic probing and small-angle X-ray scattering (SAXS) analyses revealed three conformations—an unfolded, extended conformation; a partially folded conformation stabilized by Mg^2+^; and a compact conformation stabilized by Mg^2+^ and c-di-GMP. The latter was elucidated by X-ray crystallography^16^,^18^. Guided by these studies, fluorophores were introduced into suitable locations to characterize the riboswitch by single-molecule FRET^19^ (Fig. 1a,b). The RNA was found to exist in four populations with distinct dynamic behaviours, and the relative proportions of these varied as a function of Mg^2+^ and c-di-GMP concentrations. Bulk measurements (Supplementary Table 1 and Supplementary Figs 1 and 2) show that the amplitude of the FRET change that accompanies ligand binding is dampened as Mg^2+^ ion concentration is raised above physiologic concentrations (that is*,>*1 mM). Even collectively, these various experiments only sampled a handful of solution conditions. Nonetheless, their outcomes already hint at complex conformational and energetic landscapes underlying the function of this gene-regulatory RNA. Because of its important role in bacterial physiology, the c-di-GMP riboswitch is a potentially valuable target for novel antibacterial compounds. For drug discovery, it is important to develop methods more efficient than conventional SAXS and FRET to map more comprehensively the molecular behaviour of the RNA and its response to agonists and antagonists (Fig. 1c,d).

### 3D conformational landscape of the c-di-GMP-I riboswitch

**Figure 2:**
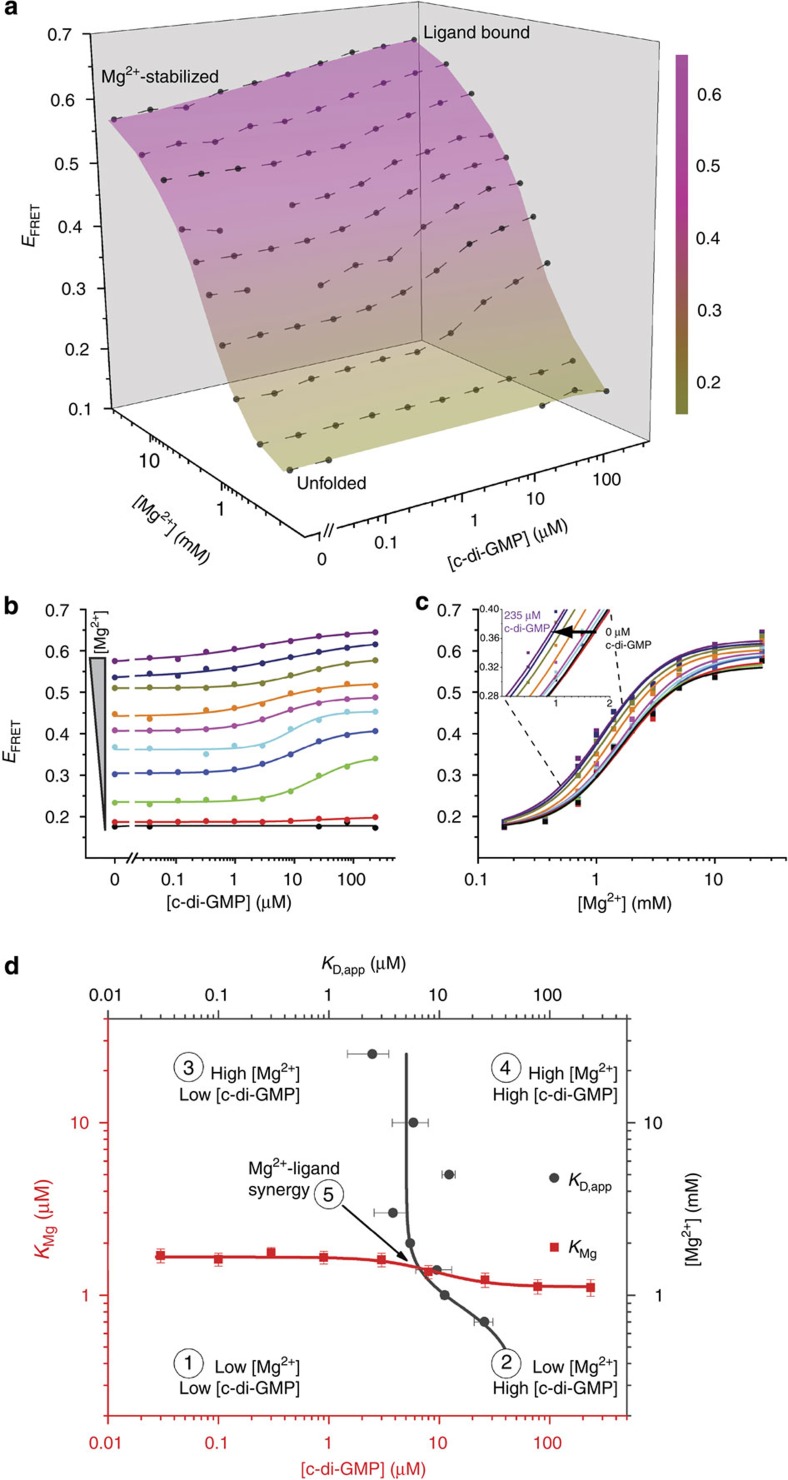
Experimentally determined RNA conformational landscape describes interaction of the c-di-GMP-I riboswitch with its cognate ligand. (**a**) Conformational landscape (coloured surface) and *E*_FRET_ data (dots with connecting lines) of the riboswitch as a function of the concentrations of Mg^2+^ and c-di-GMP, derived from a single experiment (Methods). The average standard deviation across *E*_FRET_ measurements is 0.012 and seven individual data points were removed from the data set due to spurious fluorescence measurements (Methods). The *E*_FRET_ increases in response to higher Mg^2+^ or c-di-GMP concentrations to varying degrees under all conditions probed. Full multiplexed experimental landscapes were replicated three times. One replicate is plotted here. (**b**) Data re-plotted as two-dimensional titrations of c-di-GMP at various Mg^2+^ concentrations (from 0.167 mM, black, to 25 mM, purple). The lines represent fits to each c-di-GMP titration using a two-state binding model. (**c**) Data re-plotted as two-dimensional titrations of Mg^2+^ at various c-di-GMP concentrations (from 0 μM, black, to 235 μM, purple). The lines represent fits to each Mg^2+^ titration using a semi-empirical Hill-type analysis (Methods). The inset shows the gradual shift to lower Mg^2+^ midpoint with increasing c-di-GMP concentration. (**d**) A joint plot of the midpoints of each c-di-GMP titration (*K*_D,app_, grey) and each Mg^2+^ titration (*K*_Mg_, red) describes five regimes for the response of the RNA to these two variables (see Text). The intersection of the plots (regime 5) occurs at ∼1.4 mM Mg^2+^ and ∼6.5 μM c-di-GMP. Here, the highest synergy is observed between Mg^2+^ and c-di-GMP in eliciting the RNA conformational response.

As previous work indicates that folding of the c-di-GMP-I riboswitch aptamer domain is a function of both Mg^2+^ and c-di-GMP concentrations, we sought to elucidate, in a comprehensive manner, the interaction between these two variables. To achieve this rapidly, we employed a 384-well microplate fluorometer to measure the FRET efficiency (*E*_FRET_) of the labelled c-di-GMP aptamer domain under 100 different conditions (ten concentrations of Mg^2+^ versus ten of c-di-GMP). This experiment readily (<5 min for measurements and<5 min for data processing) produced a multi-dimensional RNA conformational landscape (Methods and Fig. 2a). As Mg^2+^ or c-di-GMP concentration increased, the RNA transited from unfolded to folded conformations, albeit to widely varying degrees. Three conformational states were assigned across the 100 solution conditions, consistent with previous SAXS experiments^16^: unfolded (*E*_FRET_ ∼0.17), Mg^2+^-stabilized (*E*_FRET_ ∼0.56) and ligand-bound (*E*_FRET_ ∼0.62; ±0.012, Methods).

To facilitate evaluation, the 3D conformational landscape could be sectioned across either variable, yielding c-di-GMP or Mg^2+^ FRET titrations (Fig. 2b,c). Although extracted from a high-throughput, multi-dimensional experiment, these data are comparable in quality to those obtained from conventional 1D experiments. Comparison of FRET changes at multiple Mg^2+^ concentrations showed that this riboswitch exhibits maximal c-di-GMP binding-induced conformational change at near-physiologic Mg^2+^ concentrations^20^ (Supplementary Fig. 2). The landscape could also be visualized by plotting the midpoint of the transitions against either independent variable (Supplementary Fig. 3). Thus, a plot of c-di-GMP apparent binding affinity, *K*_D,app_, as a function of Mg^2+^ concentration demonstrated increasing affinity of the RNA for its cognate ligand up until 3 mM Mg^2+^, after which no marked enhancement in binding was observed (Supplementary Fig. 3a). The reciprocal plot of the Mg^2+^ concentration midpoints (*K*_Mg_) as a function of c-di-GMP concentrations clearly showed that the affinity of this riboswitch construct for c-di-GMP is ∼8 μM in the region (1–1.5 mM Mg^2+^) of maximal conformational response (Supplementary Figs 2 and 3b).

A joint plot of *K*_D,app_ and *K*_Mg_ for this riboswitch delineated five interaction regimes between Mg^2+^ and c-di-GMP (Fig. 2d). Inherently, the *K*_D_ or *K*_Mg_ midpoints bisect each individual titration series. Connection of these midpoints across the orthogonal variable (for example, *K*_Mg_ at various c-di-GMP concentrations or *K*_D,app_ across Mg^2+^ concentrations) subdivides the landscape into four regimes, with a fifth regime located at the intersection of these midpoints. First, at the lowest Mg^2+^ and c-di-GMP concentrations, the RNA is unfolded. Second, an increase in c-di-GMP concentrations alone produces negligible ligand binding. Third, higher Mg^2+^ concentrations produce a c-di-GMP binding-competent RNA state. Fourth, highly stabilizing Mg^2+^ concentrations dampen the conformational response to ligand binding (Fig. 2b). Fifth, at the intersection of the *K*_D_ and *K*_Mg_ plots (∼1.4 mM Mg^2+^ and ∼6.5 μM c-di-GMP) lies the regime of highest synergy between Mg^2+^ and c-di-GMP, where the highest slopes in the Mg^2+^ and c-di-GMP titrations are observed (Fig. 2b, cyan and Fig. 2c, orange). Under these conditions, ligand binding-induced conformational change, the key to riboswitch function, is maximal.

### A non-cognate ligand stabilizes a cryptic RNA conformer

As a step towards discovering non-cognate ligands that modulate the structure and function of the c-di-GMP-I riboswitch, we examined RNA–aminoglycoside interactions. Aminoglycoside antibiotics are cationic amino-sugar molecules that inhibit protein synthesis primarily by binding to the ribosomal A-site^21^,^22^. In addition, this class of compounds has been found to interact with and modulate the function of diverse non-coding RNAs^23^,^24^,^25^.

Based on the ability of several aminoglycosides to promote the folding of a three-way junction in the central domain of the 16S rRNA, it was proposed that the positively charged aminoglycosides interact with RNA primarily through nonspecific charge neutralization^26^. When we examined the effect of the aminoglycoside kanamycin B (∼200 μM) on the c-di-GMP-I aptamer domain, we initially found that at 1 mM Mg^2+^ concentration, it did not bind the RNA (unchanged *E*_FRET_), whereas at 10 mM Mg^2+^ it appeared to unfold it (Supplementary Table 2). These results would not be expected if the interaction between the riboswitch and kanamycin B were purely a nonspecific electrostatic interaction, as higher ionic strength (high Mg^2+^) should weaken the interaction between the RNA and the aminoglycoside. Moreover, if the aminoglycoside were purely a nonspecific cationic counterion to the anionic RNA, it should promote its folding (higher *E*_FRET_). Control experiments using single-fluorophore-labelled constructs ruled out the possibility that kanamycin B aberrantly affects the fluorescent properties of the dyes (Supplementary Fig. 4). Given the Mg^2+^-dependent differences in *E*_FRET_ response, we reasoned that a multiplexed analysis of this RNA–ligand interaction would better elucidate the contribution of kanamycin B to the (un)folding of this riboswitch.

**Figure 3:**
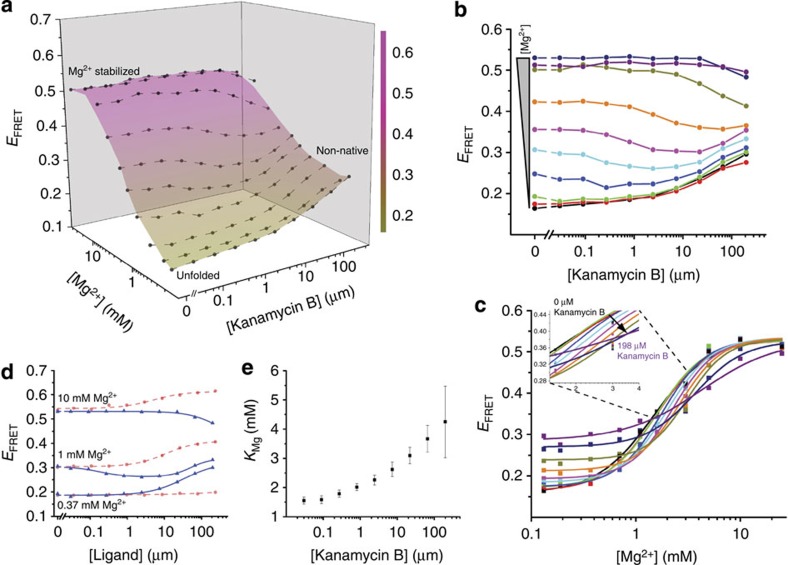
Conformational landscape analysis of c-di-GMP-I riboswitch interaction with a non-cognate ligand reveals a cryptic non-native conformation. (**a**) Conformational landscape (coloured surface) and *E*_FRET_ data (dots with connecting lines) of the riboswitch as a function of the concentrations of Mg^2+^ and the non-cognate ligand kanamycin B. *E*_FRET_ increases in response to higher Mg^2+^, but both increases and decreases in response to kanamycin B. Full multiplexed experimental landscapes were replicated four times. One replicate is plotted here. (**b**) Data re-plotted as kanamycin B titrations under various Mg^2+^ concentrations (from 0.132 mM, black, to 25 mM, purple). Lines connecting the data points do not represent fits and are visual aids only. (**c**) Data re-plotted as Mg^2+^ titrations under different kanamycin B concentrations (from 0 μM, black, to 198 μM, purple). Lines represent Hill-type fits to the Mg^2+^ titrations. The inset shows the gradual shift to higher Mg^2+^ midpoint with increasing kanamycin B concentration. (**d**) Comparison of *E*_FRET_ changes at three Mg^2+^ concentrations (low, 0.37 mM; high, 10 mM; and near physiological, 1 mM) in response to titrations of c-di-GMP (red circles, dashed line) or kanamycin B (blue triangles, solid line). The ligands induce *E*_FRET_ responses that are distinct in magnitude and sign from each other under all Mg^2+^ concentrations tested. (**e**) A plot of *K*_Mg_, obtained from fits to Mg^2+^ titrations, as a function of kanamycin B concentration. Increasing kanamycin B concentration progressively inhibits the Mg^2+^-induced folding of the RNA (higher *K*_Mg_).

To further characterize this riboswitch–aminoglycoside interaction, we determined the corresponding 3D conformational landscape (Fig. 3a), which differed notably from the landscape of the RNA in the presence of its cognate ligand (Supplementary Fig. 5). Whereas c-di-GMP binding caused monotonic *E*_FRET_ increases at nearly all measured Mg^2+^ concentrations (Fig. 2b), kanamycin B induced more complex patterns of change in the FRET readout (Fig. 3d). The landscape revealed a biphasic response of the riboswitch to kanamycin B (Supplementary Note 1). At low Mg^2+^, *E*_FRET_ increased with increasing aminoglycoside concentration, whereas in high Mg^2+^, *E*_FRET_ decreased (Fig. 3b,d; compared with Supplementary Table 2). At near-physiologic Mg^2+^ concentrations, increasing kanamycin B initially lowered but subsequently raised *E*_FRET_ (Fig. 3b, cyan and magenta, and Fig. 3d). At 1.1 mM (Fig. 3b, cyan) and 1.4 mM Mg^2+^ (Fig. 3b, magenta), *E*_FRET_ was essentially identical, within experimental error, at the lowest and highest aminoglycoside concentrations tested. The landscape exhibited a stable *E*_FRET_ state (*E*_FRET_ ∼0.30) under conditions of low Mg^2+^ (<1 mM) and high kanamycin B concentrations (Fig. 3a, lower right), a feature absent from the smooth c-di-GMP landscape (Fig. 2a). This additional *E*_FRET_ state is also apparent when individual Mg^2+^ titrations are stacked side-by-side (Fig. 3c). Analysis of these titrations revealed a trend in *K*_Mg_ as a function of kanamycin B that did not follow the corresponding trend observed in the presence of c-di-GMP (Fig. 3e and Supplementary Fig. 3b). Taken at face value, this would mean that kanamycin B inhibits the Mg^2+^-induced folding transition. The complex kanamycin B-induced riboswitch conformational landscape could indicate that the aminoglycoside both unfolds and refolds the RNA in a Mg^2+^-dependent manner, possibly populating non-native states. A competing interpretation is that increasing kanamycin B drives the RNA monotonically down a folding pathway (leading to an idiosyncratic structure) for which the mean distance between our FRET probes (positioned to report on the native folding transition) does not vary linearly. Overall, features of the kanamycin B landscape suggest the presence of structural state(s) not populated in the c-di-GMP landscape.

### 4D thermal stability landscape analysis of RNA conformers

**Figure 4:**
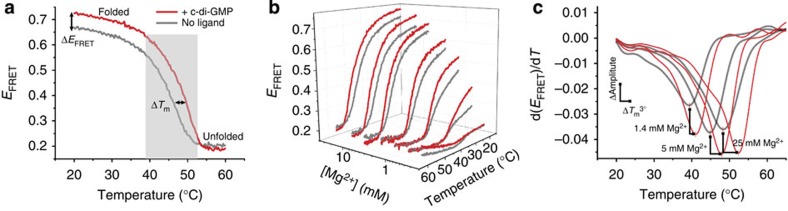
4D analysis of riboswitch conformation and tertiary structural stability in a single experiment. (**a**) Temperature-induced tertiary structure unfolding, measured using a qPCR machine, reported by change in *E*_FRET_ in the absence (grey) and presence (red) of c-di-GMP in 10 mM Mg^2+^. The RNA cooperatively unfolds as temperature increases. The addition of c-di-GMP increases both *E*_FRET_ and tertiary melting temperature (*T*_m_^3°^). (**b**) 3D plot of temperature-induced tertiary unfolding showing representative data obtained from a multiplexed experimental setup (seven Mg^2+^ concentrations, two c-di-GMP concentrations (0, grey; 235 μM, red) and 194 temperatures; Methods). (**c**) Representative plots of the *E*_FRET_ derivative of temperature-induced unfolding at three Mg^2+^ concentrations in the absence (grey) and presence (235 μM, red) of c-di-GMP. *T*_m_^3°^ is the minimum of each curve. The high quality of the data allows comparison of changes in *T*_m_^3°^ as well as the derivative amplitude, which indicates the relative fraction of folded RNA initially present in each condition.

To further compare and characterize riboswitch conformations resulting from binding to different ligands, we devised a high-throughput differential scanning FRET experiment that reports on the thermodynamic stability of the structural states (Supplementary Note 2). Like the simpler differential scanning fluorimetry assay^27^, this experiment can be performed on a repurposed qPCR instrument. We simultaneously recorded fluorescence emission from each of 100 solution conditions spanning a broad range of Mg^2+^ and ligand concentrations while varying the temperature between 20 and ≥60 °C, and generated FRET melting curves (Fig. 4 and Supplementary Figs 6, 7 and 8). From each curve, we determined the tertiary structure unfolding midpoint (Fig. 4c), or *T*_m_^3°^ (Supplementary Note 3), in effect quantifying the conformational stability of the RNA under 100 conditions. This high-content 4D experiment also revealed a putative two-step tertiary folding process for the c-di-GMP-I riboswitch that had not been previously documented (Supplementary Fig. 9).

**Figure 5:**
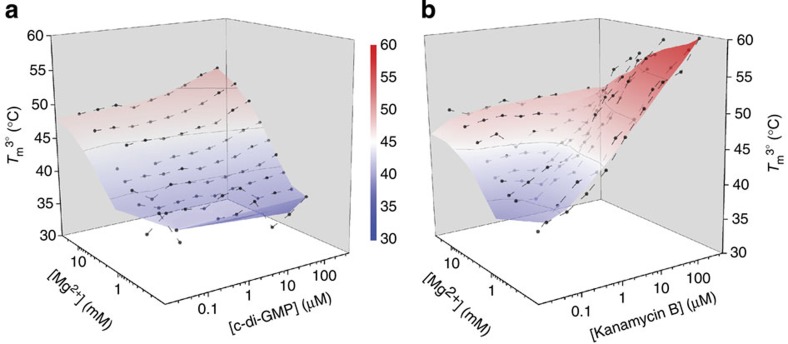
Tertiary structure stability landscapes of the c-di-GMP-I riboswitch in the presence of cognate and non-cognate ligand binding. Full multiplexed experimental landscapes were replicated three times for each ligand. One replicate of each is plotted here. (**a**) *T*_m_^3°^ landscape as a function of Mg^2+^ and c-di-GMP. Additions of Mg^2+^ or c-di-GMP both monotonically increase the *T*_m_^3°^. (**b**) *T*_m_^3°^ landscape as a function of Mg^2+^ and kanamycin B. Addition of kanamycin B does not affect the *T*_m_^3°^ at high Mg^2+^ concentrations. At low Mg^2+^ concentrations, the *T*_m_^3°^ increases in the presence of kanamycin B (see also Supplementary Fig. 10 for details). The highest *T*_m_^3°^ for this riboswitch was determined in the presence of the non-cognate ligand kanamycin B.

To facilitate analysis, we represented the data as 3D *T*_m_^3°^ landscapes over the range of Mg^2+^ and ligand concentrations (Fig. 5 and Supplementary Fig. 10) to allow side-by-side comparison with the conformational landscape for a given ligand and comparison of the respective *T*_m_^3°^ and conformational landscapes between different ligands (Supplementary Note 4). The shapes of the c-di-GMP-induced conformational and stability landscapes were similar (Figs 2a and 5a). This concordance indicated that the folding pathway of this riboswitch is free of the non-productive (that is, trapped) intermediates documented for many other RNAs^28^,^29^ (Supplementary Note 5). In this case, measurements of RNA folding and stability are interchangeable; the most folded state is also the most stable, consistent with the thermodynamic hypothesis of macromolecular folding^30^. In contrast, the shapes of the kanamycin B conformational and stability landscapes were dissimilar (Figs 3a and 5b).

Under conditions where the additional *E*_FRET_ state (∼0.30) was observed in the conformational landscape of the RNA in the presence of kanamycin B (Fig. 3a), the corresponding stability landscape exhibited the highest *T*_m_^3°^ (∼60 °C). Control experiments with singly labelled RNA constructs clearly demonstrate that *T*_m_^3°^ measurements reflect conformational stability rather than artefactual dye fluorescence in the presence of kanamycin B, over the temperature range examined (Supplementary Figs 11–13). If this *E*_FRET_ state represented a productive, partially folded conformation, a commensurately intermediate *T*_m_^3°^ would be expected in the stability landscape. However, the *T*_m_^3°^ of this kanamycin B-induced state is higher than any other *T*_m_^3°^ measured in the presence of the cognate ligand (Fig. 5a). The disparity in shape between the kanamycin B conformational and stability landscapes indicates that an alternative conformation that was not populated in the presence of c-di-GMP was indeed stabilized (Supplementary Note 5). In this case, tertiary RNA folding and stability are not concomitant because the RNA becomes trapped in an off-pathway conformation by kanamycin B.

### The kanamycin B–riboswitch interaction is specific

**Figure 6:**
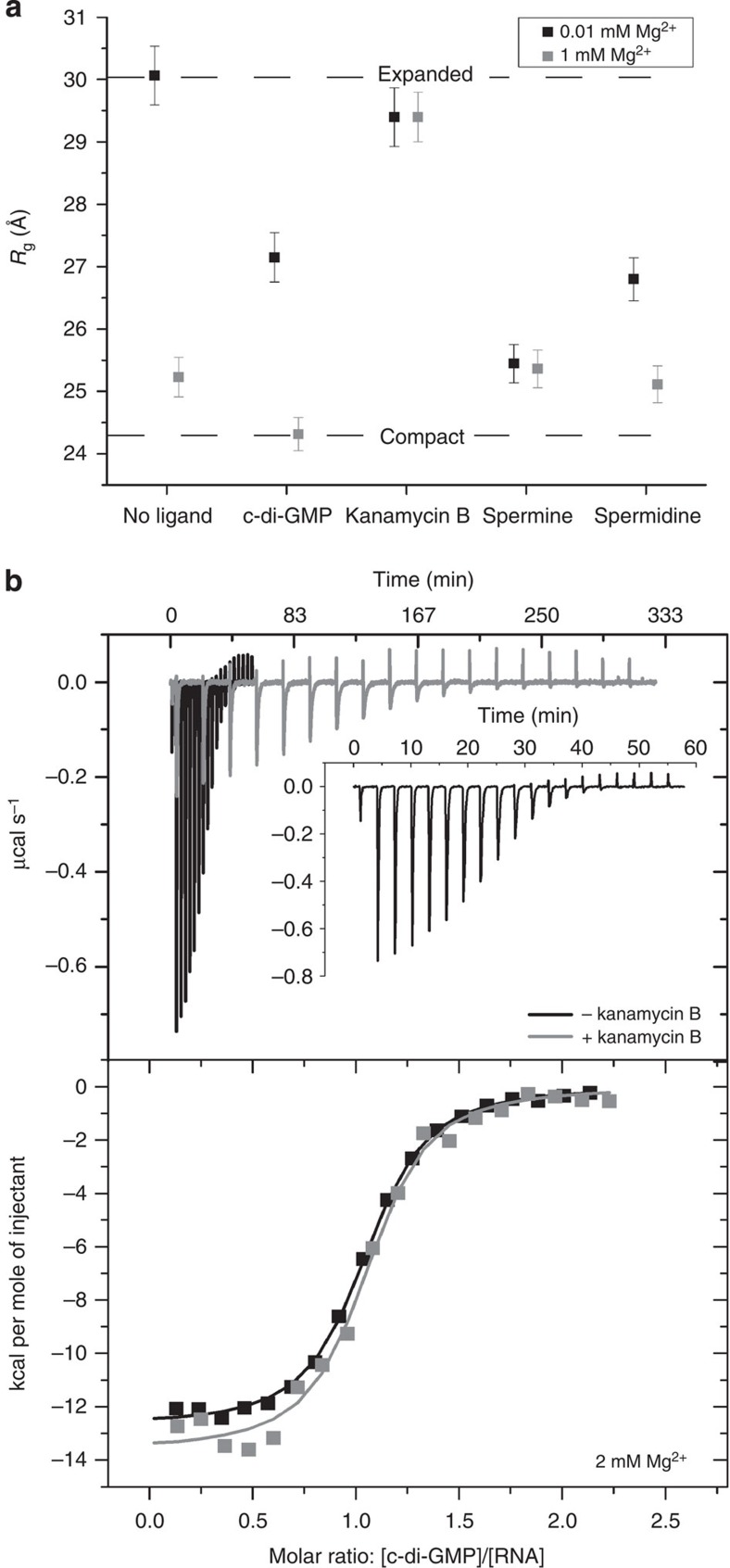
Kanamycin B induces an off-pathway conformation of the c-di-GMP-I riboswitch. (**a**) The radius of gyration (*R*_g_) of the riboswitch (measured by SAXS) in 0.01 and 1 mM Mg^2+^ (black and grey, respectively) is modulated by various ligands and polyamines. Lines marking the expanded (0.01 mM Mg^2+^, no ligand) and compact (1 mM Mg^2+^,+c-di-GMP) states clearly delineate the range of *R*_g_ in these experiments. Spermine, spermidine, c-di-GMP and Mg^2+^ promote significant compaction of the RNA; kanamycin B does not. High-quality SAXS data were recorded for each sample one time. The error in *R*_g_ is obtained from the Guinier fit. (**b**) Isothermal titration calorimetry (ITC) experiments wherein c-di-GMP (500 μM) was titrated into the riboswitch (50 μM) in a buffer containing 2 mM Mg^2+^ and either 0 μM (black) or 200 μM kanamycin B (grey). After each injection of c-di-GMP, the system equilibrates slowly when kanamycin B is present but rapidly in the absence of kanamycin B (black, and inset). The experiment is completed in less than 60 min in the absence of kanamycin B but requires nearly 333 min when kanamycin B is present. ITC experiments were performed in duplicate.

To test whether the association of kanamycin B with the riboswitch arose from nonspecific electrostatic interactions, we examined the impact of monovalent cations and polycationic amines on the conformation of the RNA. We generated riboswitch conformational and stability landscapes as a function of Mg^2+^ and monovalent cations (equimolar Na^+^ and K^+^) at up to 2 M concentration (Supplementary Fig. 14). The resulting landscapes were similar, but incongruent with the kanamycin B landscapes, suggesting that the aminoglycoside functions through a different mechanism from simple ions (Supplementary Note 5). However, it is possible that the charge density of the ligand may be more important than overall ionic strength in inducing RNA conformational change^31^,^32^. To explore this, we employed SAXS to measure the radius of gyration (*R*_g_, a measure of macromolecular size) in the presence of various ligands including the polyamines spermine and spermidine (Fig. 6a). In low Mg^2+^ conditions, the compaction induced by either spermine or spermidine (300 μM final) correlated with their respective valence and charge density; spermine (4+) compacted the RNA more than spermidine (3+). This is consistent with the activity of nonspecific charge neutralization. If the primary mode of interaction between the riboswitch and kanamycin B were nonspecific charge neutralization, at minimum similar compaction should be induced by the polycationic kanamycin B (5+) as observed in the presence of spermine and spermidine. In contrast, addition of kanamycin B did not produce large changes in *R*_g_. The RNA persisted in an expanded conformation (*R*_g_ ∼29.5 Å) that was not disrupted by the addition of up to 1 mM Mg^2+^, a condition under which (in the absence of the aminoglycoside) the RNA compacts readily (Fig. 6a). Together, the results of these experiments probing the role of ionic strength and charge density suggest that the kanamycin B–riboswitch interaction is not purely of nonspecific electrostatic nature, but that the aminoglycoside binds specifically to the RNA and stabilizes one or more non-native structures.

### Kanamycin B modulates binding of c-di-GMP to the riboswitch

We hypothesized that the non-native structure induced by kanamycin B would modulate binding of c-di-GMP to the riboswitch. We evaluated this using isothermal titration calorimetry (ITC). Pre-incubation of the riboswitch with kanamycin B resulted in a small decrease (∼1.4-fold) in c-di-GMP-binding affinity (Fig. 6b and Supplementary Table 3). Furthermore, c-di-GMP binding was more enthalpically favourable but entropically unfavourable. Similar changes were observed for ITC experiments performed in both 2 and 10 mM Mg^2+^. Overall, the presence of kanamycin B resulted in small but reproducible unfavourable changes in the free energy of c-di-GMP binding (Supplementary Table 3). Inspection of the raw ITC data revealed that, in contrast to the modest changes in thermodynamic parameters, the kinetics of c-di-GMP binding to the riboswitch were strongly affected by kanamycin B (Fig. 6b). In the absence of kanamycin B, each c-di-GMP injection equilibrated within 3 min (Fig. 6b, black, and inset). In contrast, up to 20 min were needed to reach equilibrium when the riboswitch was pre-incubated with the aminoglycoside (Fig. 6b, grey), demonstrating that c-di-GMP binding is slowed in the presence of kanamycin B. Thus, c-di-GMP binding may require structural rearrangement coupled to slow dissociation of kanamycin B from the riboswitch.

## Discussion

We have developed a FRET-based method to examine the effect of multiple environmental variables on the conformation of an RNA in a single rapid, high-throughput experiment. Our method generates empirical high-information content RNA conformational and stability landscapes using a fluorescence microplate reader and conventional qPCR instrumentation. The biophysical approach we describe here is distinctly different from and, therefore, complementary to the majority of high-information content nucleic acid structure–function characterization methods described to date, which have implemented massively parallel sequencing technologies to explore the genotype–phenotype (that is, fitness) landscapes of functional nucleic acids^33^,^34^ and nucleic acid–protein complexes^35^,^36^,^37^.

By facilitating visualization of the response of RNA conformation and stability to a broad range of experimental conditions, our multi-dimensional method readily revealed properties of the c-di-GMP-I riboswitch that could have been missed using conventional biophysical approaches. For instance, the lack of response of *E*_FRET_ to c-di-GMP at low Mg^2+^ concentration (Fig. 2), or to kanamycin B at high Mg^2+^ concentration (Fig. 3), might have led to the erroneous conclusions that neither ligand interacts with the RNA. Instead, our experimental landscapes indicate that under those particular divalent cation concentrations, the RNA is either too unfolded or folded, respectively, to bind to the small molecules. Indeed, under conditions commonly employed to examine riboswitch–ligand interactions (for example, >20 mM Mg^2+^), the riboswitch–kanamycin B interaction would not have been identified using conventional ligand titration or thermal melting assays (Supplementary Note 6). Our 4D FRET method demonstrates that correct characterization of RNA ligands is highly contingent on solution conditions. Low-dimensional single-point or ligand titration assays can be insufficient to identify or characterize known and novel RNA–ligand interactions. Hence, our method, which uncovered regimes of distinct Mg^2+^-ligand synergy, strategically informs the design of quantitative high-throughput screening^38^ assays targeting RNA. Moreover, comparison of conformation and tertiary structure stability landscapes between different ligands immediately suggested the presence of otherwise hidden, alternative RNA conformations capable of ligand binding.

The stabilization of a non-native conformation of the c-di-GMP-I riboswitch aptamer domain induced by kanamycin B binding, which we have characterized by 4D FRET, SAXS and ITC, suggests new paradigms for pharmacological targeting of small molecule-responsive RNAs. By analogy with how kanamycin B alters the binding of the cognate ligand to the riboswitch, it may be fruitful to search for new ligands that inhibit RNA function by binding non-competitively. It is now widely appreciated that many riboswitches are under kinetic, rather than thermodynamic, control^39^,^40^,^41^. Kinetic competition for binding with the cognate ligand, resulting from stabilization of off-pathway conformations, represents a new potential avenue for the discovery of antibiotic leads targeting bacterial riboswitches.

Our high-information content method employs instrumentation that is commonly available in many research environments (Supplementary Note 7), and can be readily extended to evaluate additional factors (for example, time, pH, interacting ligands) influencing RNA structure and function. We therefore anticipate that our multiplexed FRET approach will be useful in characterizing RNA–protein interactions^42^,^43^,^44^,^45^ or RNAs with known alternative conformations^46^,^47^, and for the development of high-throughput screening assays targeting such systems.

## Methods

### RNA preparation

The c-di-GMP-I riboswitch aptamer domain constructs (Supplementary Fig. 1) used in this study are derived from the *tfoX* 5'-UTR of *Vibrio cholerae* and incorporate the point mutation G20U^48^. The mutant RNA binds c-di-GMP with low micromolar affinity, and was chosen to facilitate the identification of weakly binding competitive ligands. The full-length aptamer domain construct (96 nt), transcribed *in vitro* and purified as described^49^, was used for SAXS and ITC. Briefly, RNA was transcribed from a PCR template and purified using denaturing PAGE. The appropriate band was excised and the RNA was then electroeluted (Elutrap, GE) overnight. The RNA was concentrated and buffer exchanged with 1 M KCl once followed by exchange with water three times using centrifugal ultrafiltration (Amicon Ultra, 10k). RNA was stored at −20 °C. For fluorescence experiments, a bimolecular RNA construct identical to that previously described^19^, except for the G20U mutation, was employed. Briefly, two synthetic RNA oligonucleotides (Yale University Keck Foundation Biotechnology Resource Facility) were deprotected^50^ and purified by denaturing PAGE. The RNAs were electroeluted, and concentrated and washed with water by centrifugal ultrafiltration (Amicon Ultra, Millipore). Purified RNAs were stored at −20 °C. Cy3 was incorporated during synthesis into the red strand (Supplementary Fig. 1). The blue strand contained an amine linker for labelling with Cy5. This RNA was labelled overnight as described^50^, ethanol precipitated and then labelled a second time to increase labelling efficiency. Following ethanol precipitation, the RNA was resuspended in 5% (v/v) acetonitrile and 95% (v/v) 50 mM triethylammonium acetate (pH 7) before loading onto a C-18 reversed-phase HPLC column (XDB-C18, Agilent) equilibrated in the same buffer. A 15-column volume gradient from 5 to 40% acetonitrile at a flow rate of 3 ml min^−1^ was used for purification. Typically, 15 nmoles of RNA were separated in a 9.4 × 250 mm^2^ column. Labelling efficiency was estimated to be 70–85% from chromatogram integration. The purified, labelled RNA was frozen (−80 °C), lyophilized, resuspended in water and stored at −20 °C.

The bimolecular RNA used for fluorescence measurements was prepared as follows. A 200 μl mixture of 5 μM Cy5-containing RNA and 7.5 μM Cy3-containing RNA were incubated overnight in folding buffer (25 mM NaCl, 25 mM KCl, 20 mM HEPES-KOH, pH 7.5) containing 2 mM MgCl_2_ and fractionated on a 24-ml bed-volume Superdex 75 (GE) size-exclusion chromatography column equilibrated in the same buffer. The bimolecular RNA (30 kDa) was well separated from the excess Cy3-containing RNA (10.2 kDa). Absorbance was recorded at 260, 540 and 640 nm to monitor RNA, Cy3 and Cy5, respectively. The areas under the Cy3 and Cy5 curves pertaining to the bimolecular peak were compared and the Cy5:Cy3 molar ratio was determined (1.02:1,±0.01 Cy5 molar excess).

### Samples for multiplexed FRET experiments

Folding buffers (20 mM HEPES-KOH, pH 7.5, 20 mM NaCl, 20 mM KCl) containing ten different MgCl_2_ concentrations were prepared. A unique folding buffer was dispensed into each of ten columns in a 384-well microplate; the same Mg^2+^ folding buffer was present in ten rows of each column. Dual-labelled RNA was added to each of the 100 wells to a final concentration of 25 nM. For experiments containing c-di-GMP, 250 nl of a different concentrated ligand stock solution were dispensed to each of the ten rows using a mosquito Crystal (TTP Labtech). This resulted in final concentrations across the rows ranging from 0.1 to 25 mM Mg^2+^ and down the columns ranging from 0 to 235 μM c-di-GMP in a final volume of 21.25 μl. The microplate was covered with an aluminum seal, centrifuged for 1 min at 700 r.p.m., and incubated on an orbital shaker at 200 r.p.m. overnight at room temperature. For experiments containing kanamycin B, the RNA was incubated overnight in the absence of ligand. Then, 250 nl of each ligand stock were dispensed using a mosquito Crystal (TTP Labtech) as described above to yield a 10 × 10 matrix ranging from 0.1 to 25 mM Mg^2+^ and from 0 to 198 μM kanamycin B in a final volume of 25.25 μl. The microplate was covered with an aluminum seal, centrifuged for 1 min and incubated on an orbital shaker at 200 r.p.m. for 2.5 h. To evaluate the fully unfolded RNA, EDTA was added to a final concentration of 4 mM to additional RNA samples containing 0.1 mM Mg^2+^.

### Samples for tertiary thermal melts on qPCR machine

Samples for thermal unfolding experiments were prepared similarly to the method described above for multiplexed FRET measurements. 20 μl of the samples containing c-di-GMP (described above) were transferred to a 384-well qPCR plate and centrifuged at 1,000 r.p.m. for 1 min before measurement on the qPCR machine. Samples containing kanamycin B were prepared as described above with slight modifications. The RNA was incubated in the presence of ligand overnight on an orbital shaker (200 r.p.m.) at a final concentration of 25 nM RNA in 25.25 μl. The plate was centrifuged for 1 min before measurement.

Higher noise in *E*_FRET_ measurements was observed for data obtained on the qPCR machine, likely due to the sensitivity of the optical readout and background signal from the qPCR plate. The higher noise in the *E*_FRET_ signal precluded fitting the Mg^2+^ titration and c-di-GMP titration data at room temperature. However, the determination of *T*_m_^3°^ was robust when compared with samples prepared at various concentrations and volumes of RNA. An increase in RNA concentration and volume (40 nM, 25 μl) did allow for robust measurements of *E*_FRET_ on the qPCR machine with reduced noise and of sufficient quality to fit two Mg^2+^ titrations (in the absence and presence of c-di-GMP). The fits were in agreement with measurements made on the microplate fluorometer.

### Fluorescence measurements and *E*_FRET_ landscapes

Room temperature fluorescence measurements were recorded using a Tecan Infinite 200 Pro microplate reader with a single excitation filter corresponding to Cy3 excitation (535±12.5 nm) and two emission filters corresponding to Cy3 and Cy5 emissions (595±17.5 and 670±12.5 nm). The apparent FRET efficiency^50^,^51^ was calculated using *E*_FRET_=*I*_A_/(*I*_D_+*I*_A_). Origin 9.1 software (OriginLab) was used to generate smoothed 3D surface landscapes. The *E*_FRET_ data from the 10 Mg^2+^ titrations were smoothed using adjacent-averaging with a window of five points (Origin 9.1). The smoothed data were plotted as a 3D surface and coloured according to the range of *E*_FRET_ values. Seven data points from the c-di-GMP landscape were removed because of significantly lowered Cy3 and Cy5 reads, suggestive of insufficient volume in the well. From the kanamycin B landscape, the *E*_FRET_ at 25 mM Mg^2+^ was unexpectedly lower than the *E*_FRET_ at 10 mM Mg^2+^. The data in all other Mg^2+^ concentrations were robust and in agreement with Mg^2+^ titrations performed on other days. A disproportionate loss of signal of both Cy3 and Cy5 intensities at 25 mM Mg^2+^ was responsible for the lower *E*_FRET_ and was attributed to sample adherence to the microcentrifuge tube used during sample preparation of this sample.

### Tertiary thermal unfolding measurements and *T*_m_^3°^ landscapes

A ViiA7 (Applied Biosystems) qPCR machine was used to simultaneously record melting curves for the RNA tertiary structure under each solution condition from 20 to 70 °C using a ramp rate of 2.5 °C min^−1^. Cy3 and Cy5 (via FRET) emissions were measured 194 times over this range and a FRET melting curve was generated using one excitation filter 520±10 nm (Cy3) and two emission filters at 586.5±10 nm (Cy3) and 682±14 nm (Cy5). Changes in fluorescence signal outside the cooperative tertiary unfolding transition were observed because of photobleaching and the temperature-dependent quantum yields of the dyes. The *E*_FRET_ thermal unfolding curves (194 data points) were smoothed using an FFT filter using a 10-point window and cutoff frequency of 0.193 (Origin 9.1). Derivatives of the smoothed *E*_FRET_ thermal unfolding curves were calculated and peaks corresponding to *T*_m_^3°^ were identified using Origin 9.1 (OriginLab). The *T*_m_^3°^ landscape surface was generated by smoothing the Mg^2+^ titration *T*_m_^3°^ data using adjacent averaging (Origin 9.1).

### Preparation and measurement of singly labelled RNAs

Singly labelled RNA constructs were prepared and purified identically to dual-labelled constructs. Explicitly, the Cy3-labelled oligo (red, Supplementary Fig. 1) was annealed to a RNA strand of identical sequence to the blue strand (Supplementary Fig. 1) but without a fluorophore attached. Similarly, the Cy5-labelled oligo (blue, Supplementary Fig. 1) was annealed to a RNA strand of identical sequence to the red strand (Supplementary Fig. 1) but lacking a fluorophore. Following purification by size-exclusion chromatography, as described above, samples were prepared as follows. Folding buffers (20 mM HEPES-KOH, pH 7.5, 20 mM NaCl, 20 mM KCl) containing ten different MgCl_2_ concentrations were prepared. Each sample contained 23 μl of buffer and 2 μl of RNA. Samples were prepared in triplicate. Following overnight incubation in the Mg^2+^-containing buffers, 1 μl of ligand stock or water was added. Final concentrations were 25 nM RNA in 10 Mg^2+^ concentrations between 0.17 and 25 mM Mg^2+^. Each Mg^2+^ titration was performed in 0 and 200 μM kanamycin B. Samples were incubated for 1.5 h followed by recording fluorescence emissions using a Tecan M1000pro. For Cy3, the excitation wavelength used was 535±20 nm and the emission wavelength was 595±20 nm. For Cy5, the excitation wavelength used was 635±20 nm and the emission wavelength was 670±20 nm. Subsequently, 21 μl of each sample were transferred to a 384-well qPCR plate and data were recorded following a total incubation time of 5 h in the presence of ligand. Thermal measurements employed a 2.5 °C min^−1^ ramp rate and the following filter sets: Cy3 singly labelled RNA, 520±10 nm (excitation) and 586±10 nm (emission); Cy5 singly labelled RNA, 640±10 nm (excitation) and 682±14 nm (emission).

### Data analysis and fitting of titration curves

All Mg^2+^ titrations were fit using a semi-empirical Hill-type analysis described previously^52^. The signal of the unfolded state was fixed at *E*_FRET_=0.17, in accordance with that observed in the presence of 4 mM EDTA. The c-di-GMP titrations were fit to a two-state binding model to obtain *K*_D,app_. In addition, the c-di-GMP titrations were fit using a Hill analysis to obtain measures of the slope of each titration.

### *E*_FRET_ error analysis and data scaling

Slightly different *E*_FRET_ values for Mg^2+^ titrations were observed for samples prepared on different days. This difference was likely due to variations in the molar ratio of the purified bimolecular construct or photobleaching of one of the dyes during sample preparation. Despite different *E*_FRET_ values, similar *K*_Mg_ parameters were obtained from fits to the Mg^2+^ titrations. Furthermore, nearly identical *T*_m_^3°^ were measured from the thermal unfolding curves, suggesting that the unfolded and folded states were identical, despite the minor variations observed in *E*_FRET_. To establish the inherent error in *E*_FRET_ measurements, data prepared on different days were compared and the Mg^2+^ titrations in the absence of ligand were fit using a semi-empirical Hill equation. Next, the *E*_FRET_ values were normalized using the equation *S*_n_=(*S*−*S*_low_)/(*S*_high_−*S*_low_), where *S*_n_ is the normalized signal, *S* is the observed signal, *S*_low_ is the signal of the unfolded RNA and *S*_high_ is the signal of the folded RNA. *S*_low_ for all experiments was fixed to match the unfolded *E*_FRET_ value obtained in 4 mM EDTA. Using data normalized from ten experiments performed on different days over the period of several months the average standard deviation across all *E*_FRET_ values was calculated (±0.012). The error was largest (±0.02) near the Mg^2+^ midpoint (∼1–2 mM), where small changes in the [Mg^2+^] due to pipetting error result in slightly larger *E*_FRET_ differences. In the low and high end of the curve, the standard deviation is ±0.01. Using the normalized parameters, the data in the kanamycin B landscape were scaled to match the *E*_FRET_ range of the c-di-GMP landscape.

### UV melting experiments

A bimolecular riboswitch construct, identical to that used in fluorescence experiments except lacking the fluorophores, was purified by size-exclusion chromatography as described for preparation of the labelled RNA. A 750-μl purified sample of the bimolecular riboswitch RNA was prepared in a folding buffer with final concentrations of 10 μg ml^−1^ RNA, 25 mM NaCl, 25 mM KCl and 20 mM HEPES-KOH, pH 7.5 with either 2 or 10 mM Mg^2+^. Thermal melting was performed on an Agilent 8543 spectrophotometer over a temperature gradient from 20 to 90 °C in 0.1 °C increments with a 12-s equilibration at each temperature before absorbance measurements. Absorbance (260 nm) and derivative data curves were smoothed using a Savitzky-Golay 41-point filter (Origin 9.1).

### Small-angle X-ray scattering experiments

A 200-μl sample of unimolecular riboswitch RNA was prepared in folding buffer with final concentrations of 615 μM RNA, 25 mM NaCl, 25 mM KCl, 3 mM MgCl_2_ and 20 mM HEPES-KOH, pH 7.5. The higher Mg^2+^ concentration was used in this folding buffer (3 mM MgCl_2_) in order to compensate for the high concentration of RNA^52^. The RNA sample was equilibrated at room temperature for >16 h. A Superdex 200 (GE) size exclusion chromatography column was pre-equilibrated with folding buffer containing 1 mM Mg^2+^. The RNA was purified on the column and the RNA concentration was determined before preparation of samples for SAXS experimentation. SAXS samples in 1 mM Mg^2+^ were prepared by mixing 35 μl of purified RNA (1.98 g l^−1^) with 63.5 μl of the size-exclusion chromatography buffer. To prepare SAXS samples in 0.01 mM Mg^2+^, 350 μl of the purified RNA were concentrated to a final concentration of 6.7 g l^−1^ using an Amicon Ultra centrifugal filter (Millipore). 10 μl of this RNA stock were mixed with 88.5 μl of folding buffer containing 0 mM Mg^2+^. Buffer samples were prepared identically to RNA sample preparation but with the addition of appropriate folding buffer instead of RNA stock. Samples were shipped to the Advanced Photon Source with frozen cold packs. At the ID-12 BESSRC beamline, the samples were centrifuged briefly before addition of 1.5 μl of appropriate ligand (20 mM stock). The final concentration of each ligand (c-di-GMP, kanamycin B, spermine, spermidine) was 300 μM. The final RNA concentrations were 0.69 and 0.67 g l^−1^ for the samples in 1 and 0.01 mM Mg^2+^, respectively. The samples were incubated at room temperature for >11 h before data collection. Twenty consecutive exposures of 1 s were recorded while the sample was continuously flowed. Data were collected for buffer samples without RNA immediately before samples containing RNA. Data were processed using Igor Pro (Wavemetrics) as described elsewhere^53^.

### ITC experiments

The unimolecular riboswitch construct at a final concentration of 45 μM in 500 μl was mixed with folding buffer containing 2 or 10 mM MgCl_2_ and 0 or 200 μM kanamycin B (four total samples). An excess of the four folding buffers containing Mg^2+^ and kanamycin B at the aforementioned concentrations was prepared for concentration and washing of the RNA samples. The samples were concentrated tenfold using Amicon Ultra centrifugal filter units (Millipore) and washed with appropriate folding buffer. This process was repeated ≥5 times to thoroughly equilibrate the RNA in the appropriate buffers. The equilibrated samples were incubated overnight at room temperature. The following day the RNA concentrations were determined using absorbance measurements at 260 nm, including a multiplicative factor of 1.35 to correct for hypochromicity. The RNA samples were diluted in the appropriate folding buffers to achieve a final concentration of 50 μM RNA. The c-di-GMP titrant was prepared to a final concentration of 500 μM by adding 0.6 μl of 50 mM c-di-GMP to 59.4 μl of appropriate folding buffer. Hence, the buffers of the sample and the titrant were not more than 99.0% identical and a minor endothermic signal was observed as the titration curves became saturated with ligand. Raw ITC data were exported and processed using NITPIC^54^ and Sedphat^55^. The first two data points from each experiment were removed because of large residuals to the initial fit. ITC experiments were performed in duplicate.

## Additional information

**How to cite this article:** Baird, N. J. *et al.* Rapid RNA–ligand interaction analysis through high-information content conformational and stability landscapes. *Nat. Commun.* 6:8898 doi: 10.1038/ncomms9898 (2015).

## Supplementary Material

Supplementary Information Supplementary Figures 1-13, Supplementary Tables 1-3, Supplementary Notes 1-7 and Supplementary References
