# Supplementary material for: Rapid RNA–ligand interaction analysis through high-information content conformational and stability landscapes
Source: Nat Commun. 2015 Dec 7;6:8898. doi: 10.1038/ncomms9898 (PMC4686816; doi:10.1038/ncomms9898)
Supplement: Supplementary Information — Supplementary Figures 1-13, Supplementary Tables 1-3, Supplementary Notes 1-7 and Supplementary References [file ncomms9898-s1.pdf]

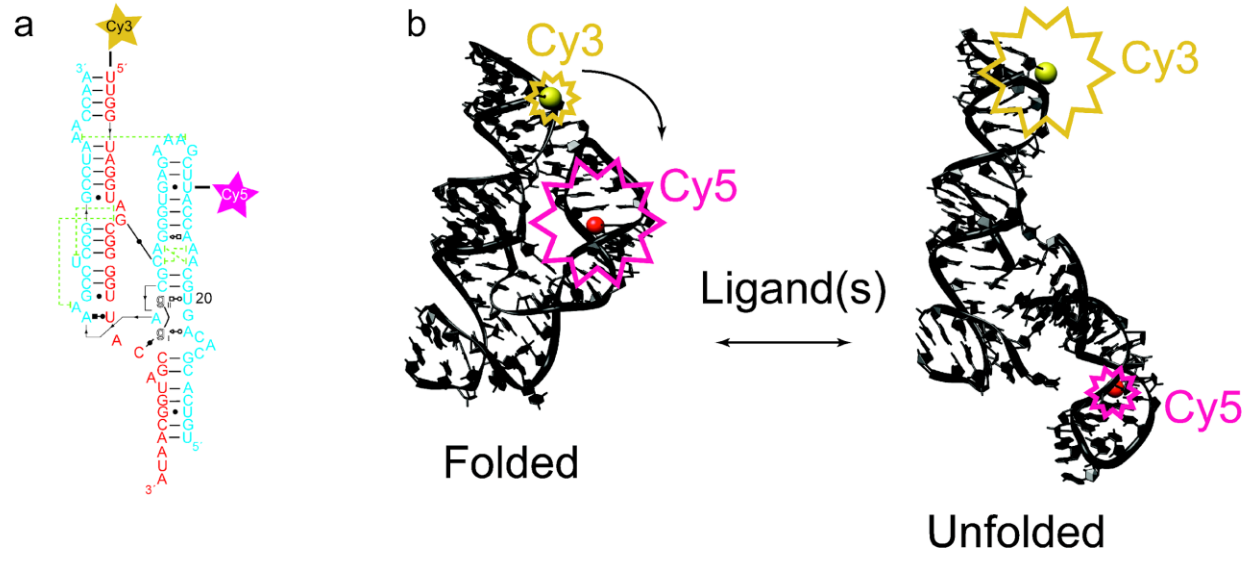

Supplementary Figure 1. Structural models and fluorophore locations. (**a**) Secondary structure diagram depicting the two-strand construct of the c-di-GMP riboswitch used in this study[1]. Green dotted lines represent stacking interactions. The cognate ligand is represented by black outlined lowercase letters. The red strand contains a Cy3 dye on its 5′ end. The blue strand contains a modified base to which Cy5 is covalently attached. This riboswitch construct incorporates a G20U mutation[2], which results in approximately micromolar c-di-GMP binding affinity (**Supplementary Table 3**). (**b**) Structural models of the riboswitch in the folded and unfolded conformations, derived from X-ray crystallography (folded) and small-angle X-ray scattering data (unfolded)[3]. In the folded conformation, the dyes are brought into close proximity, giving rise to high Förster energy transfer efficiency (E_{FRET}). The unfolded conformation exhibits low E_{FRET} due to the longer distance between the dyes.

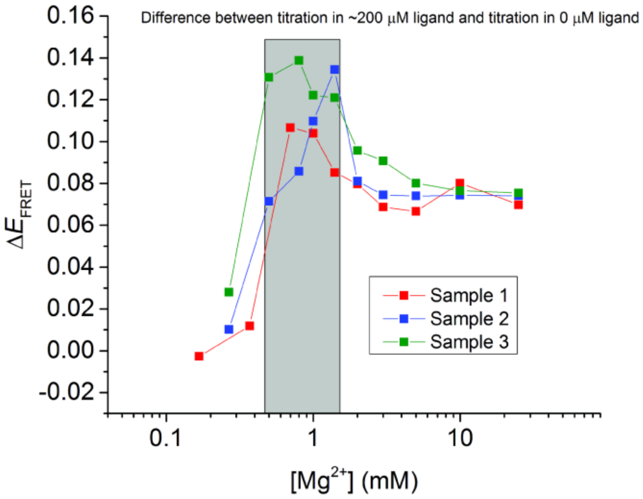

Supplementary Figure 2. Plot of ΔE_{FRET} across Mg^{2+} concentrations. The ΔE_{FRET} was calculated by subtracting the E_{FRET} signal in the absence of ligand from the E_{FRET} signal in the presence of ~200 μM c-di-GMP at each Mg^{2+} concentration tested. Data were obtained from different samples prepared on different days and were normalized based on their the unfolded and folded signals (**Methods**). The grey box highlights the maximal ΔE_{FRET} between the titrations (+/- ligand), which occurs around 1 mM Mg^{2+}.

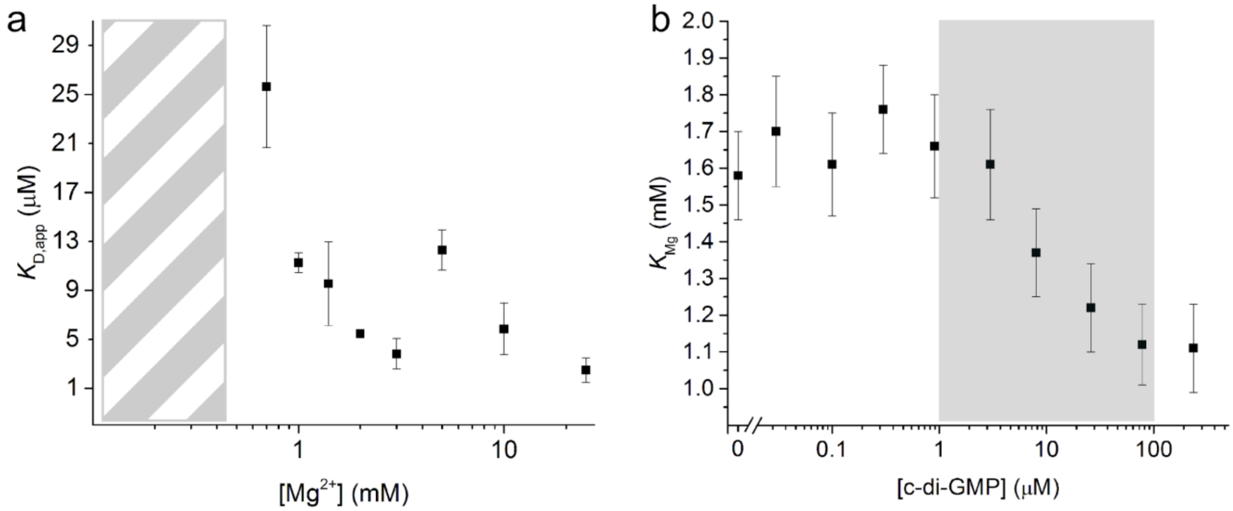

Supplementary Figure 3. Midpoints of Mg^{2+} and c-di-GMP titrations characterize the K_{Mg} and $K_{D,app}$ as a function of the orthogonal variable. (**a**) A plot of the c-di-GMP binding affinity, or $K_{D,app}$, as a function of Mg^{2+} concentration. The $K_{D,app}$ is obtained from a fit to the titration using a two-state binding model (**Methods**). The error reported is the standard error of the fit. Data at the lowest Mg^{2+} concentrations were not fit due to a low signal-to-noise ratio of the E_{FRET} data (patterned box). (**b**) A plot of the Mg^{2+} midpoint, or K_{Mg}, as a function of c-di-GMP concentration. The K_{Mg} is obtained using a semi-empirical Hill-type fit of each Mg^{2+} titration series (**Methods**). The grey box represents the 50 – 100 fold range over which the riboswitch is most responsive to changes in c-di-GMP concentration. Reported error bars are obtained from fits to the data in **Fig. 2b, c**.

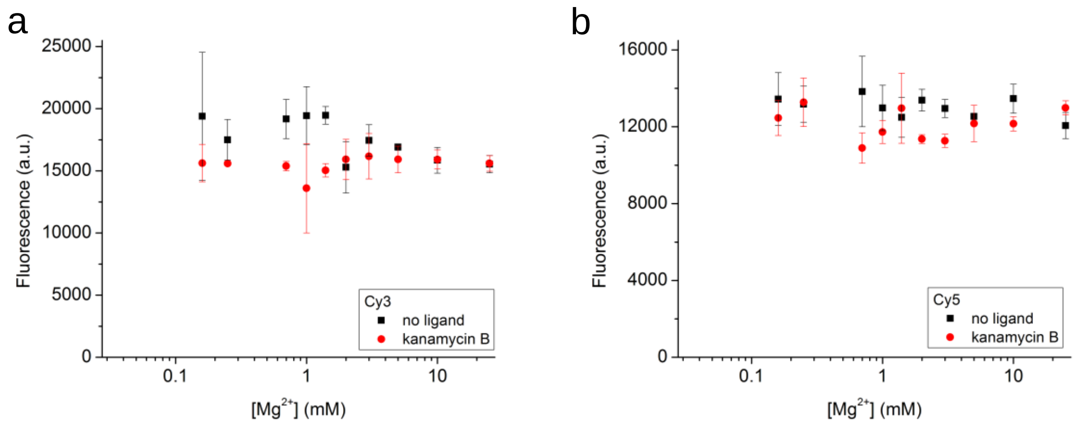

Supplementary Figure 4. RNA constructs labeled with a single fluorophore (Cy3, left panel; Cy5, right panel) reveal similar fluorescence intensities across a wide range of [Mg^{2+}] in the absence and presence of 200 μM kanamycin B. This demonstrates that the fluorescence properties of the dyes under these conditions are not altered by the RNA conformation or the presence of kanamycin B. Each experiment was performed in triplicate and is reported as the mean ± standard deviation.

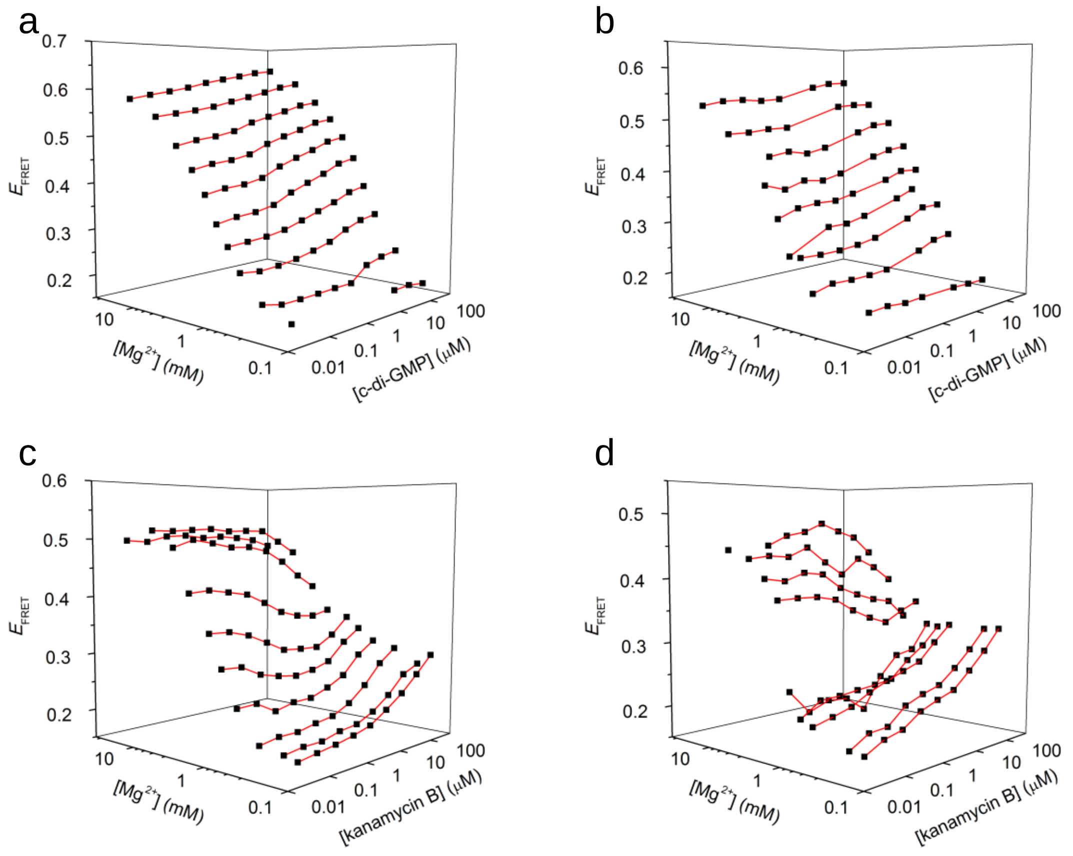

Supplementary Figure 5. Conformational landscapes are reproducible over multiple experiments. Key features of the landscape are observed in landscapes generated from samples prepared on different days, including the kanamycin B-induced increase in E_{FRET} in low Mg^{2+} concentrations and decrease in E_{FRET} in high Mg^{2+} concentrations. The Mg^{2+} and kanamycin B conditions were varied in each experiment, precluding direct merging of the data. (**a**) Conformational landscape in the presence of cyclic di-GMP, as depicted in **Fig. 2**. (**b**) Conformational landscape in the presence of cyclic di-GMP generated from separate samples prepared on a different day than in (**a**). (**c**) Conformational landscape in the presence of kanamycin B, as depicted in **Fig. 3**. (**d**) Conformational landscape in the presence of kanamycin B generated from separate samples prepared on a different day than in (**c**).

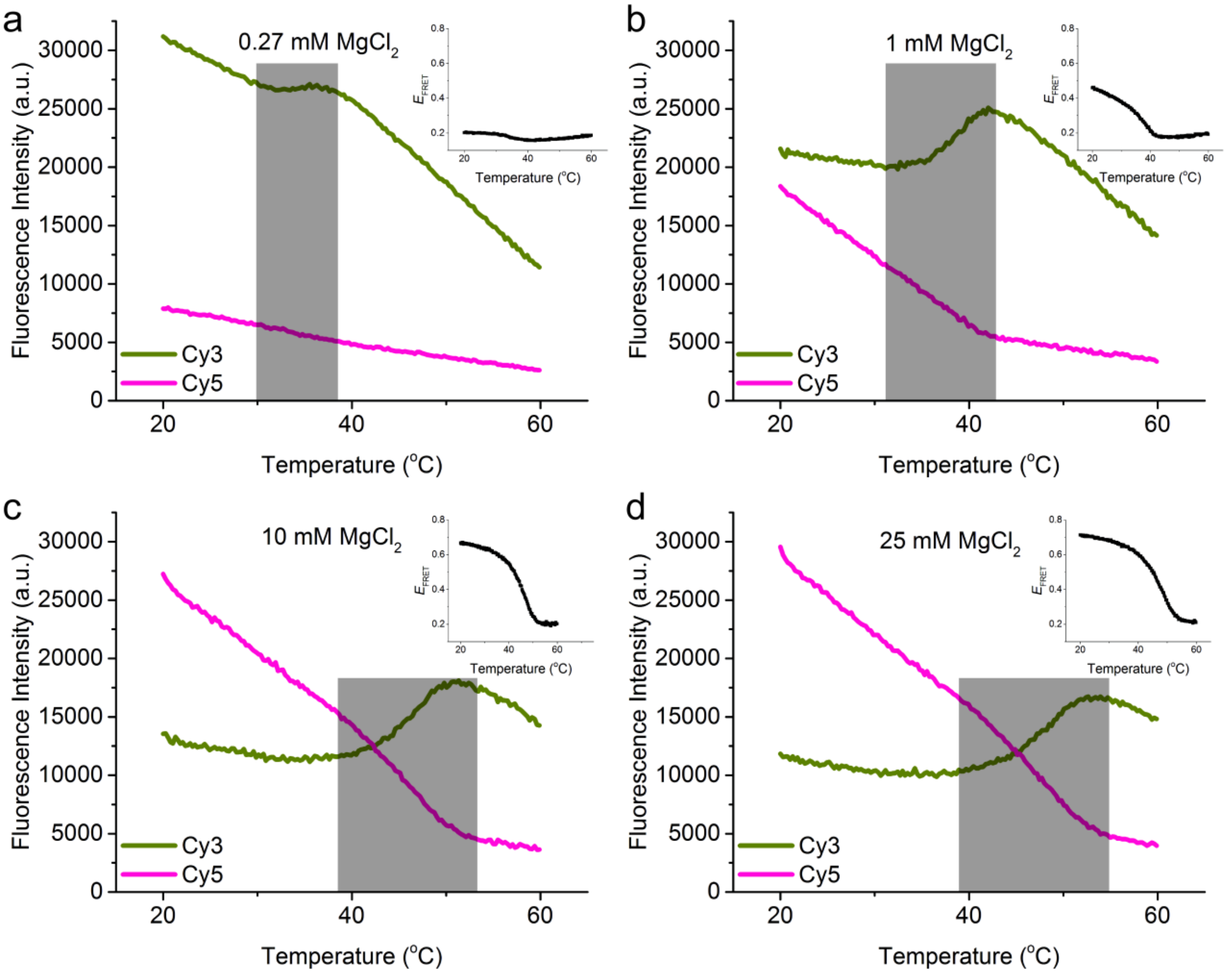

Supplementary Figure 6. Anti-correlated Cy3 and Cy5 signal changes during cooperative temperature-induced tertiary unfolding. Signal changes are plotted from samples in (**a**) 0.27 mM Mg^{2+}, (**b**) 1 mM Mg^{2+}, (**c**) 10 mM Mg^{2+}, and (**d**) 25 mM Mg^{2+}. Insets show the E_{FRET} calculated from the Cy3 and Cy5 signal intensities across temperature (**Methods**). Unfolded RNA places the dyes far apart and very little energy transfer occurs, resulting in high donor emission (Cy3) and low acceptor emission (Cy5). Folded RNA brings the dyes in close proximity and results in low donor emission (Cy3) and high acceptor emission (Cy5) due to energy transfer. The grey shaded area in each plot represent the cooperative tertiary unfolding transition. The unfolding transition spans larger temperature ranges as the RNA is stabilized in higher Mg^{2+}. Following cooperative tertiary unfolding, the changes in Cy3 and Cy5 signals are dominated by photobleaching and the temperature-dependence of the quantum yield.

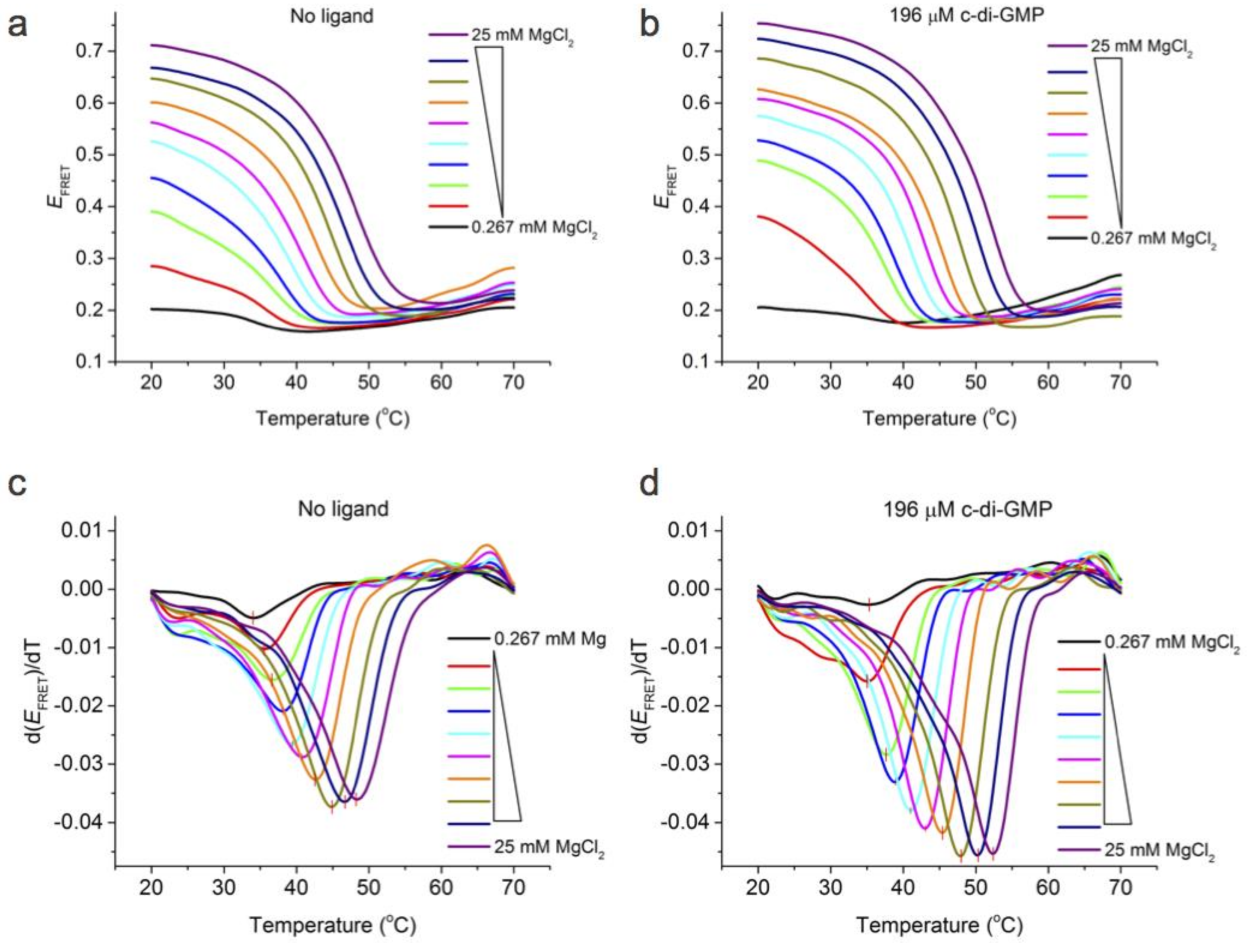

Supplementary Figure 7. Temperature-induced tertiary unfolding as a function of Mg^{2+} and c-di-GMP. Samples were prepared in 10 different Mg^{2+} concentrations (all in mM; 0.267, black; 0.5, red; 0.8, green; 1, blue; 1.4, cyan; 2, magenta; 3, orange; 5, olive; 10, navy; 25, purple). Representative E_{FRET} data (**a**) in the absence of ligand and (**b**) in the presence of 196 μM c-di-GMP were smoothed with an FFT filter using a 10-point window and cutoff frequency of 0.193 (Origin 9.1). Derivative plots of the data (**c**) in the absence of ligand and (**d**) in the presence of 196 μM c-di-GMP. The $T_m^{3°}$ is determined from the negative peak of the derivative plots and is marked by a vertical red dash. The $T_m^{3°}$ increases with increasing Mg^{2+} concentration and in the presence of c-di-GMP. The derivative amplitudes are larger in the presence of c-di-GMP than in the absence of c-di-GMP due to the increased raw E_{FRET} signal of the c-di-GMP-bound conformation.

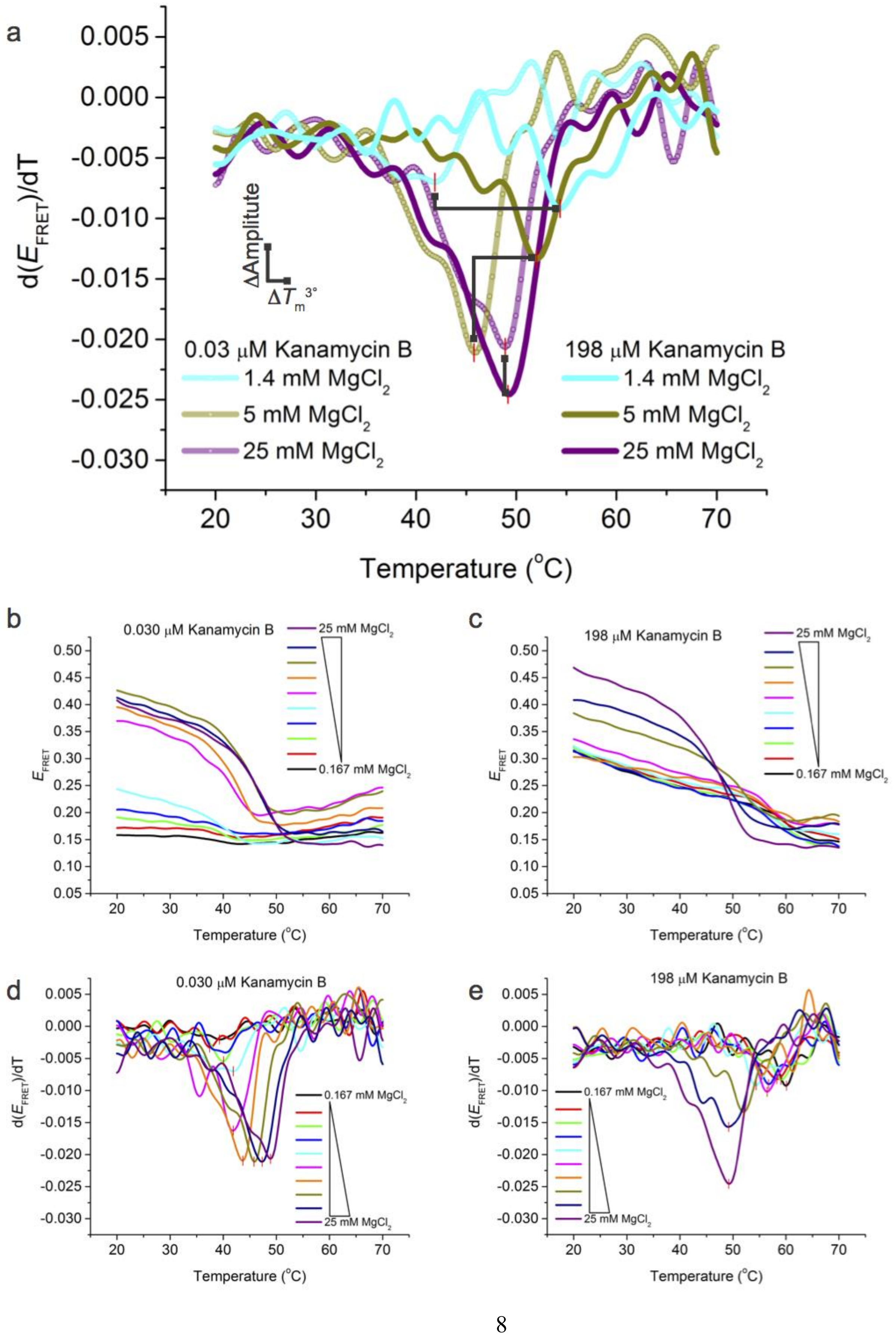
a
d(E_{FRET})/dT
Temperature (°C)
ΔAmplitute
ΔT_m 3°
0.03 μM Kanamycin B
1.4 mM MgCl2
5 mM MgCl2
25 mM MgCl2
198 μM Kanamycin B
1.4 mM MgCl2
5 mM MgCl2
25 mM MgCl2
b
0.030 μM Kanamycin B
25 mM MgCl2
0.167 mM MgCl2
E_{FRET}
Temperature (°C)
c
198 μM Kanamycin B
25 mM MgCl2
0.167 mM MgCl2
E_{FRET}
Temperature (°C)
d
0.030 μM Kanamycin B
0.167 mM MgCl2
25 mM MgCl2
d(E_{FRET})/dT
Temperature (°C)
e
198 μM Kanamycin B
0.167 mM MgCl2
25 mM MgCl2
d(E_{FRET})/dT
Temperature (°C)

Supplementary Figure 8. Temperature-induced tertiary unfolding modulated by the non-cognate ligand kanamycin B. Samples were prepared in 10 different Mg^{2+} concentrations (all in mM; 0.167, black; 0.25, red; 0.7, green; 1, blue; 1.4, cyan; 2, magenta; 3, orange; 5, olive; 10, navy; 25, purple). (a) Comparison of E_{FRET} derivative plots at low (0.03 μM, dotted line) and high (198 μM, solid line) kanamycin B concentrations in three different Mg^{2+} concentrations. The changes in amplitude and $T_{\mathrm{m}}^{3°}$ are marked by the black lines and squares. At low Mg^{2+} concentrations (e.g. 1.4 mM, blue) the addition of 198 μM kanamycin B increases the $T_{\mathrm{m}}^{3°}$ by more than 10 ºC. The $\Delta T_{\mathrm{m}}^{3°}$ is ~5 ºC in 5 mM Mg^{2+} (olive) and nearly unchanged in 25 mM Mg^{2+} (purple). The E_{FRET} data (**b**) in 0.03 μM and (**c**) 198 μM kanamycin B were smoothed with an FFT filter using a 10-point window and cutoff frequency of 0.193 (Origin 9.1). Derivative plots of the data (**d**) in 0.03 μM and (**e**) 198 μM kanamycin B with $T_{\mathrm{m}}^{3°}$ marked by a vertical red dash at the negative maximum. Comparison of panels (**d**) and (**e**) reveal that the $T_{\mathrm{m}}^{3°}$ changes induced by kanamycin B are largest at low Mg^{2+} concentrations but are nearly unchanged at high Mg^{2+} concentrations.

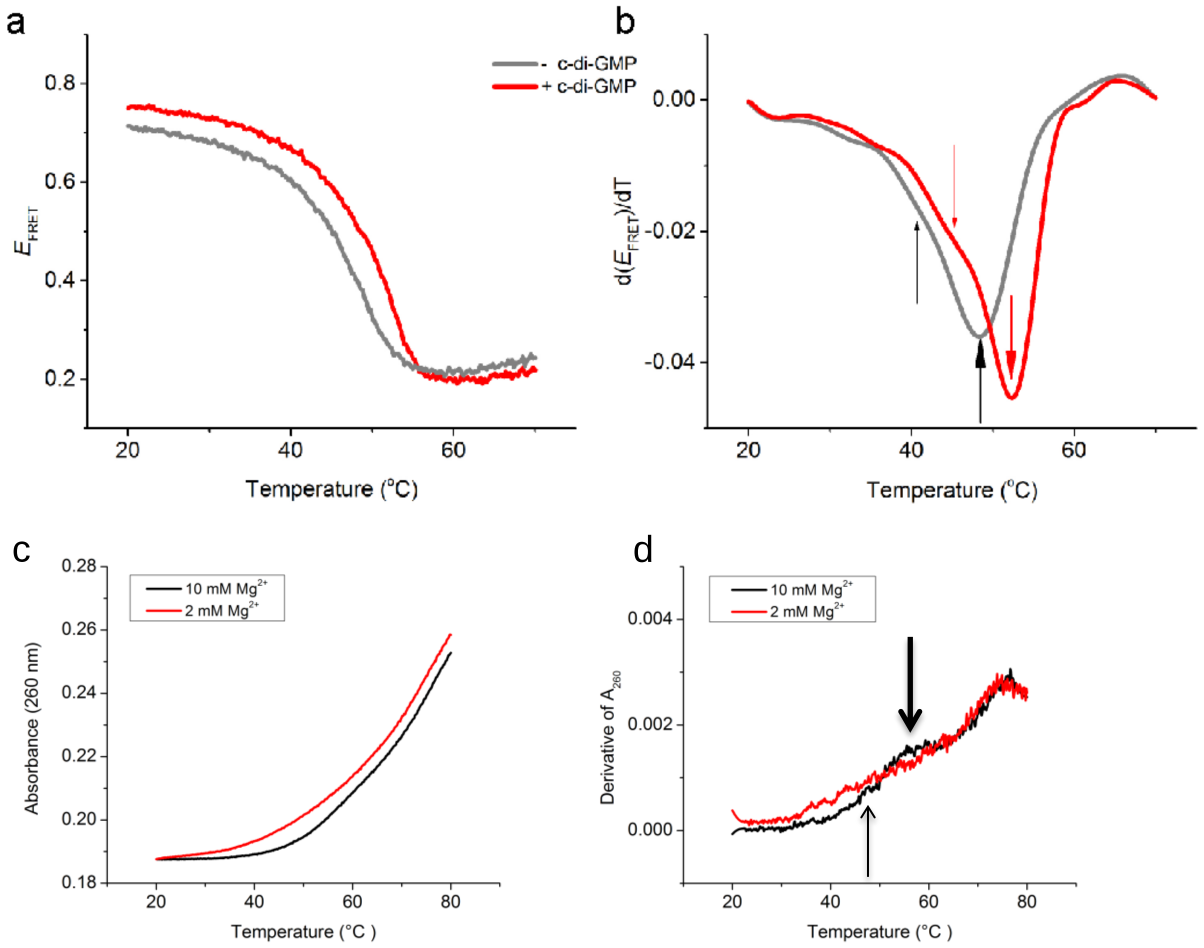

Supplementary Figure 9. Temperature-induced tertiary unfolding may involve two steps. (**a**) Temperature-induced tertiary unfolding of the bimolecular, dual-labeled c-di-GMP riboswitch construct in 25 mM Mg^{2+} monitored by FRET in the absence (black) and presence (red) of c-di-GMP. (**b**) Derivative of the E_{FRET} signal plotted in the absence (black) and presence (red) of c-di-GMP. The $T_m^{3°}$ for each curve is indicated with a thick arrow while a minor, putative unfolding step is indicated with a thin arrow. This initial unfolding event is inferred by the departure from a single Gaussian bell-shape in the derivative plot. (**c**) Thermal unfolding can also be monitored by absorbance at 260 nm using a bimolecular construct lacking fluorophores but otherwise identical to that used in the FRET assay. (**d**) Secondary structure unfolding (duplex melting) occurs at ~74 °C in 2 mM $MgCl_2$ (red), ~78 °C in 10 mM $MgCl_2$ (black). The A_{260} derivative reproducibly exhibits two small features within the tertiary unfolding peak, denoted with arrows (~48 ºC and ~56 ºC) in 10 mM Mg^{2+}. These features may correspond to a two-step tertiary unfolding process.

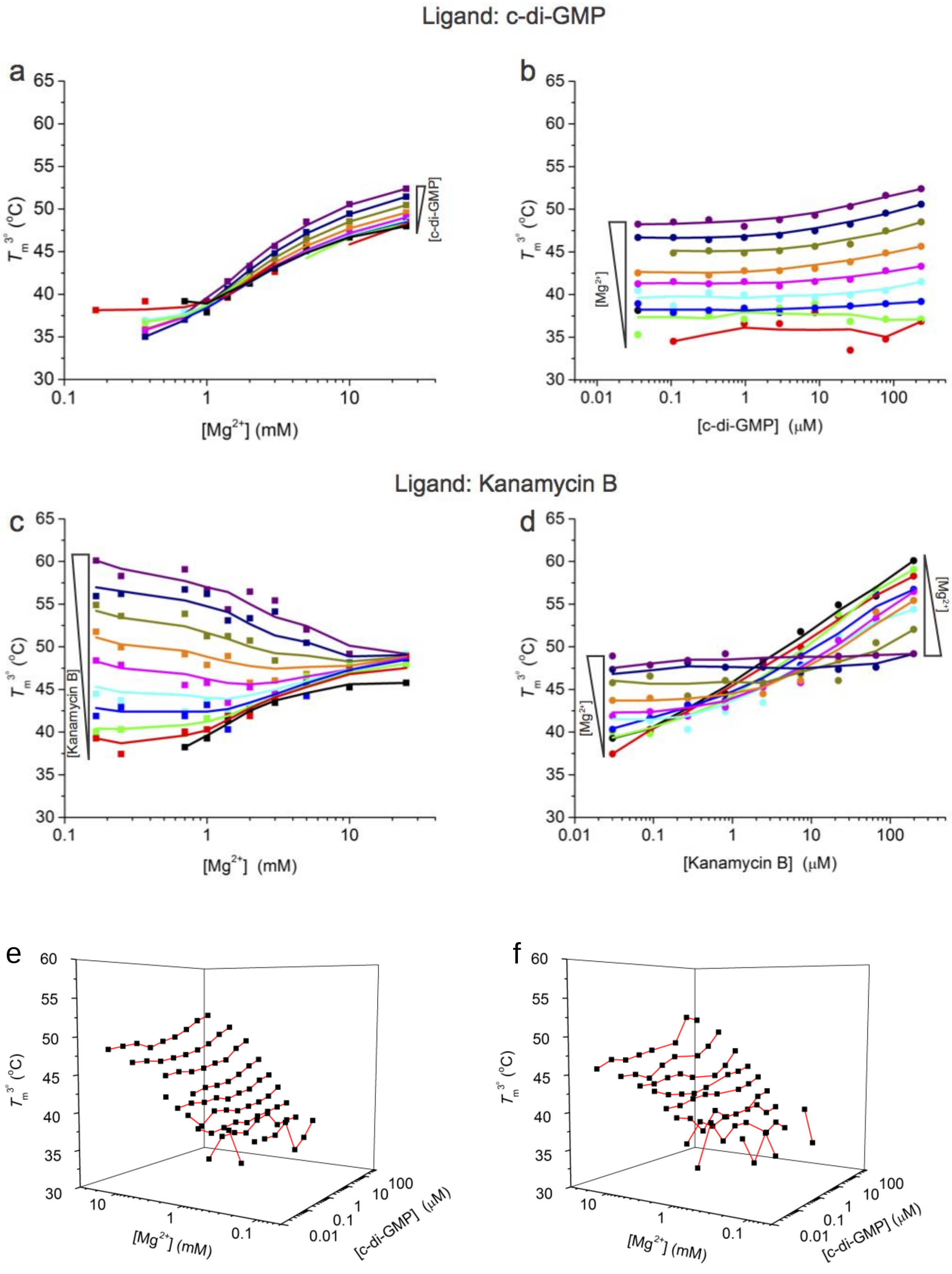
Ligand: c-di-GMP
a
b
$T_m^{3°}$ (°C)
[c-di-GMP]
[Mg2+] (mM)
[Mg2+]
[c-di-GMP] (μM)
Ligand: Kanamycin B
c
d
[Kanamycin B]
[Mg2+] (mM)
[Kanamycin B] (μM)
e
f
[Mg2+] (mM)
[c-di-GMP] (μM)

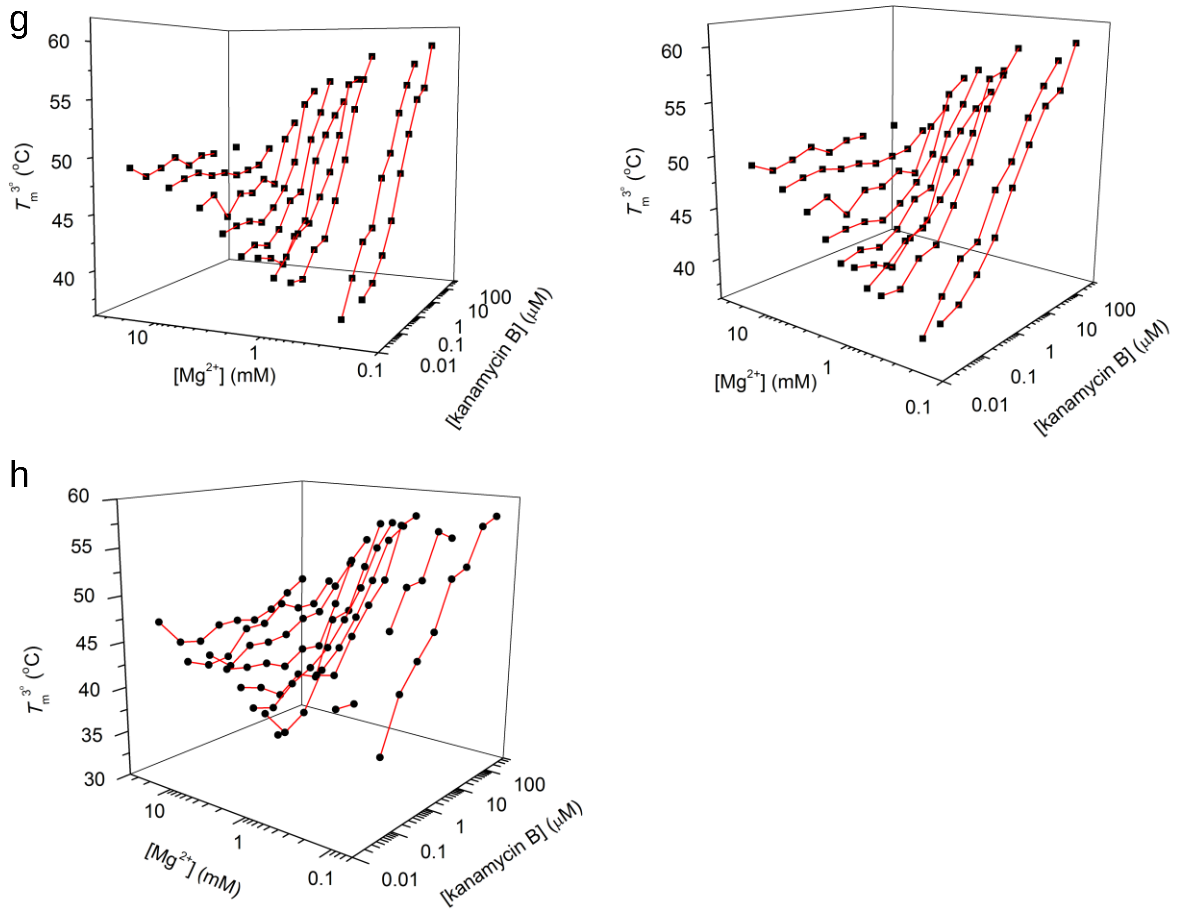

Supplementary Figure 10. Two-dimensional and three-dimensional plots of $T_m^{3°}$ as a function of Mg^{2+} and ligand. Lines represent data smoothed using Adjacent-Averaging (Origin 9.1). (**a**) $T_m^{3°}$ as a function of Mg^{2+} titration in the presence of different c-di-GMP concentrations (all in μM; 0, black; 0.035, red; 0.11, green; 0.32, blue; 0.96, cyan; 2.9, magenta; 8, orange; 26, olive; 78, navy; 235, purple). (**b**) $T_m^{3°}$ as a function of c-di-GMP titration in the presence of different Mg^{2+} concentrations (all in mM; 0.37, red; 0.7, green; 1, blue; 1.4 cyan; 2, magenta; 3, orange; 5, olive; 10, navy; 25 purple). In all Mg^{2+} concentrations that supported c-di-GMP binding the $T_m^{3°}$ increased in the presence of c-di-GMP. (**c**) $T_m^{3°}$ as a function of Mg^{2+} titration in the presence of different kanamycin B concentrations (all in μM; 0, black; 0.03, red; 0.09, green; 0.27, blue; 0.81, cyan; 2.4, magenta; 7.3, orange; 22, olive; 66, navy;

198, purple). The $T_m^{3°}$ in the presence of kanamycin B was highly dependent on Mg^{2+} concentration. At low Mg^{2+} concentrations, addition of high kanamycin B concentrations increased the $T_m^{3°}$ significantly. At high Mg^{2+} concentrations, the addition of high kanamycin B concentrations did not change the $T_m^{3°}$, within experimental error, suggesting that an RNA conformation is stabilized that is not competent to bind kanamycin B. (**d**) $T_m^{3°}$ as a function of kanamycin B titration in the presence of different Mg^{2+} concentrations (all in mM; 0.167m black; 0.25, red; 0.7, green; 1, blue; 1.4 cyan; 2, magenta; 3, orange; 5, olive; 10, navy; 25 purple). In 25 mM Mg^{2+} the $T_m^{3°}$ was identical in the absence and presence of kanamycin B suggesting that the thermal stability was not even affected by the non-specific positive charge of the aminoglycoside. Stability landscapes in the presence of c-di-GMP (**e**) from the experiment depicted in **Fig. 5a** and (**f**) from an experiment prepared with different samples on a different day are reproducible across the range of experimental conditions. The Mg^{2+} and kanamycin B concentration ranges are slightly different between the two experiments, precluding direct averaging of the data. In both landscapes, the data at lowest Mg^{2+} concentrations exhibit larger variations in $T_m^{3°}$ as a result of very low ΔE_{FRET} signal during thermal unfolding. (**g**) Multiple orientations of the stability landscape depicted in **Fig. 5b** reveal the overall shape and demonstrate the overall incongruence with the conformational landscape shown in **Fig. 3a**. Stability landscapes in the presence of kanamycin B from experiments prepared with different samples on different days are reproducible, as shown in (**g**) and (**h**). The Mg^{2+} and kanamycin B concentration ranges are slightly different between the two experiments, precluding direct averaging of the data.

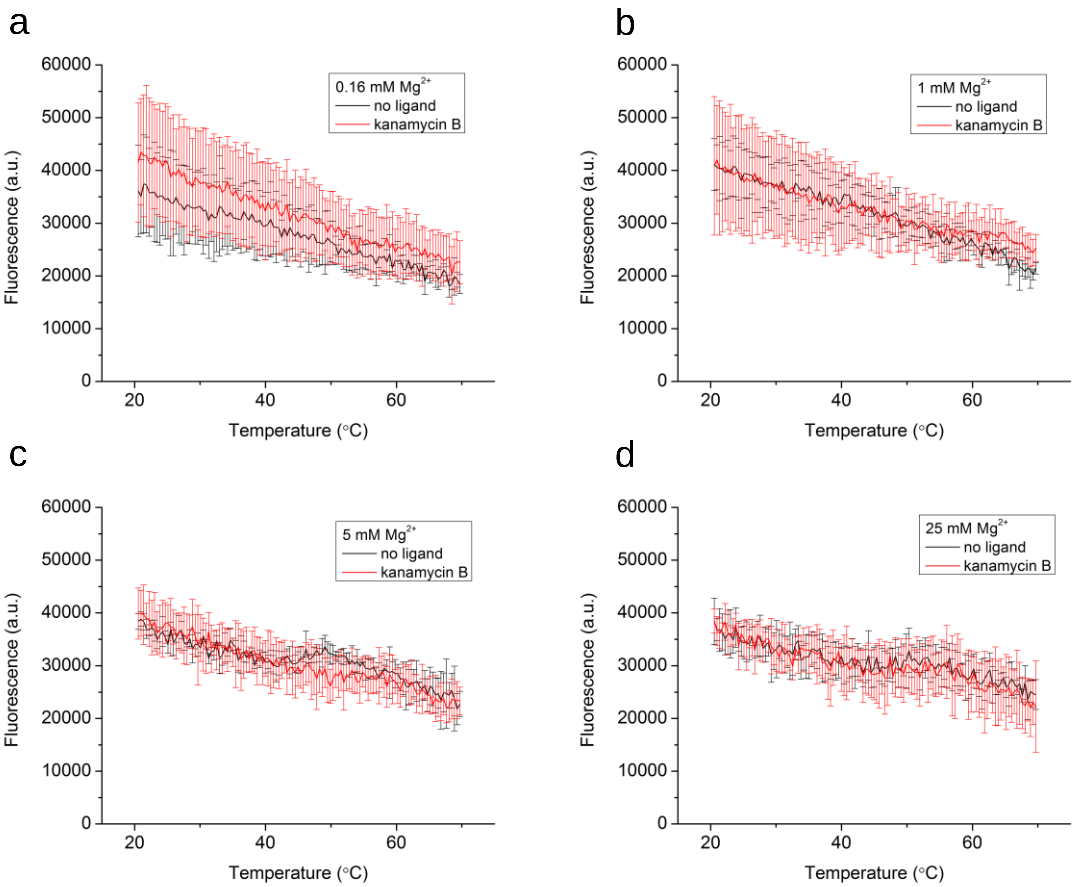

Supplementary Figure 11. Temperature-dependent fluorescence of Cy3-labeled RNA. Within experimental error, the fluorescence of a Cy3-labeled bimolecular RNA construct is identical in the absence and presence of 200 μM kanamycin B. This demonstrates that kanamycin B does not alter the fluorescence properties of Cy3 across the temperature range in (**a**) 0.16 mM Mg^{2+}, (**b**) 1 mM Mg^{2+}, (**c**) 5 mM Mg^{2+}, and (**d**) 25 mM Mg^{2+}. The bimolecular RNA construct used in this experiment is identical to that used in the FRET experiments except only the Cy3 fluorophore is incorporated. Each experiment was performed in triplicate and is reported as the mean ± standard deviation.

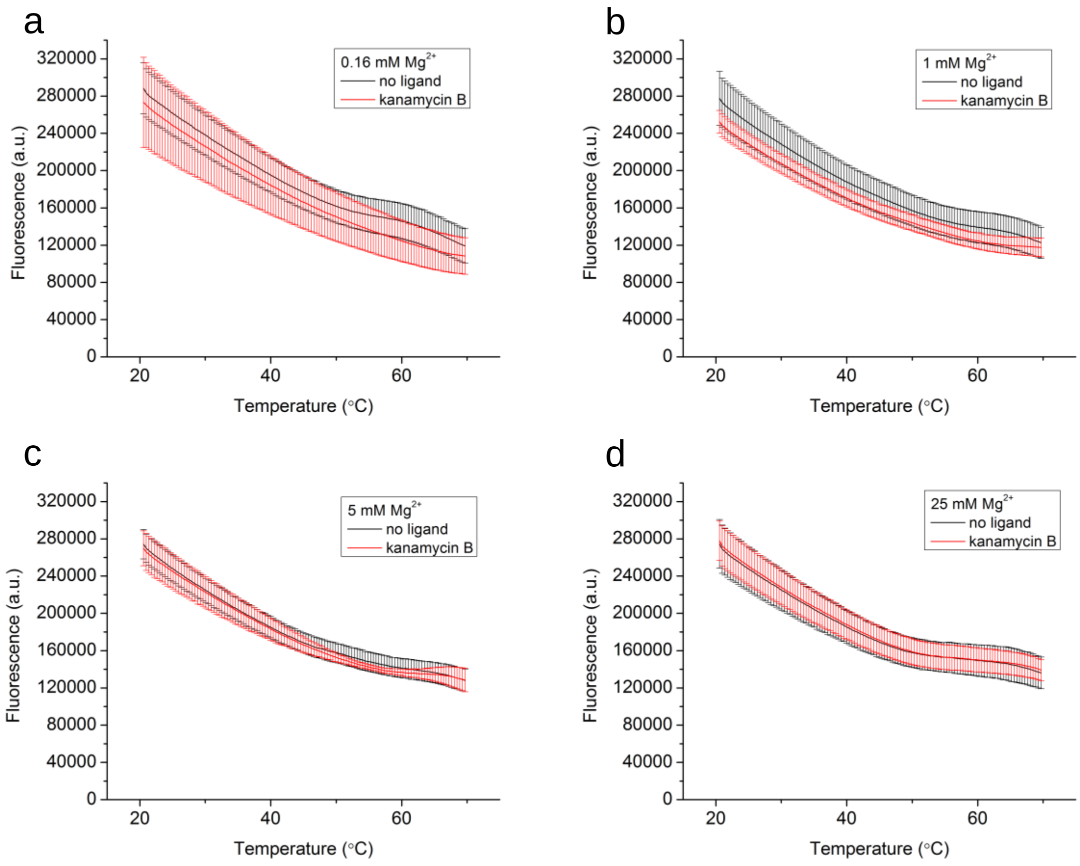

Supplementary Figure 12. Temperature-dependent fluorescence of Cy5-labeled RNA. Within experimental error, the fluorescence of Cy5-labeled bimolecular RNA construct is identical in the absence and presence of 200 μM kanamycin B. This demonstrates that kanamycin B does not alter the fluorescence properties of Cy5 across the temperature range in (**a**) 0.16 mM Mg^{2+}, (**b**) 1 mM Mg^{2+}, (**c**) 5 mM Mg^{2+}, and (**d**) 25 mM Mg^{2+}. The bimolecular RNA construct used in this experiment is identical to that used in the FRET experiments except that only the Cy5 fluorophore is incorporated. Each experiment was performed in triplicate and is reported as the mean ± standard deviation.

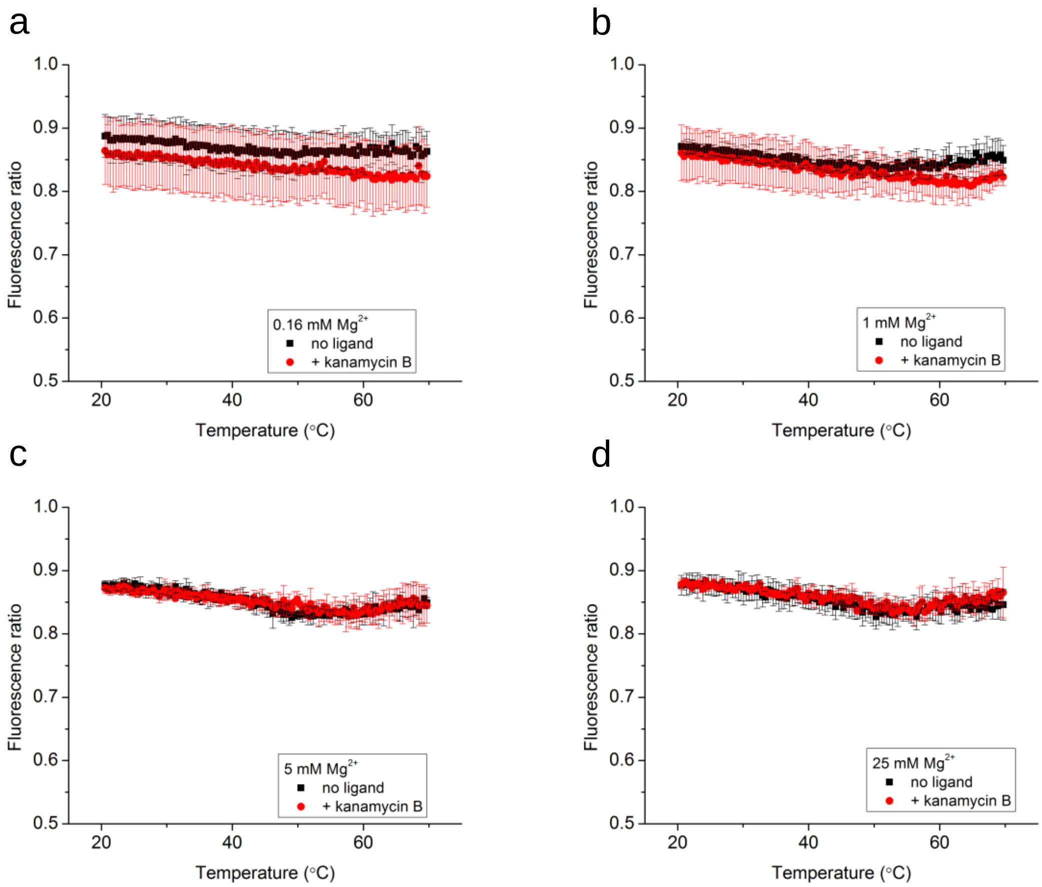

Supplementary Figure 13. Temperature-dependent fluorescence ratio. A pseudo-E_{FRET} [Cy5/(Cy5+Cy3)] was calculated from the two singly-labeled RNAs (**Supplementary Figs. 11** and **12**). Within error, the pseudo-E_{FRET} is identical in the absence and presence of 200 μM kanamycin B in (**a**) 0.16 mM Mg^{2+}, (**b**) 1 mM Mg^{2+}, (**c**) 5 mM Mg^{2+}, and (**d**) 25 mM Mg^{2+}. Generally, the pseudo-E_{FRET} decreases slightly and monotonically over the temperature range. At high [Mg^{2+}], the pseudo E_{FRET} increases slightly above ~60 °C. The slight changes in pseudo-E_{FRET} from these control experiments do not complicate interpretation of the E_{FRET} changes in the doubly-labeled RNA (**Supplementary Figs. 7** and **8**), wherein the observed E_{FRET} decreases cooperatively, not monotonically, upon unfolding, even at temperatures near 60 °C (**Supplementary Fig. 8c**). Each calculated pseudo- E_{FRET} was performed in triplicate and is reported as the mean ± standard deviation.

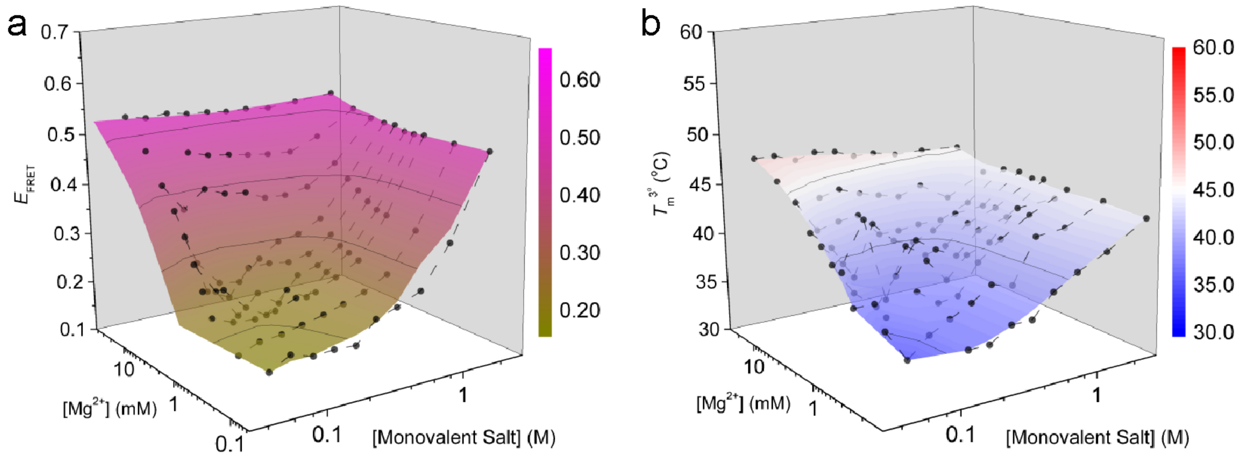

Supplementary Figure 14. E_{FRET} and $T_m^{3°}$ landscapes of the c-di-GMP riboswitch as a function of increasing ionic strength. (**a**) The E_{FRET} landscape demonstrates that both Mg^{2+} and monovalent salts (equimolar Na^+ and K^+) promote the folded conformation. (**b**) The shape of the $T_m^{3°}$ landscape is similar to the E_{FRET} landscape and indicates that charge neutralization by up to 2 M monovalent salts does not result in an increased $T_m^{3°}$ beyond that obtained with Mg^{2+} alone. Comparison of these landscapes with those obtained in the presence of kanamycin B (**Figs. 3** and **5**) indicate that charge neutralization is not solely responsible for the alternative conformation stabilized by kanamycin B.

Supplementary Tables

Supplementary Table 1. Single point E_{FRET} in the absence and presence of c-di-GMP

Ligand:	none	c-di-GMP
1 mM Mg^{2+} (± 0.02)	0.30	0.41
10 mM Mg^{2+} (± 0.01)	0.53	0.62

Supplementary Table 2. Single point E_{FRET} in the absence and presence of kanamycin B

Ligand:	none	Kanamycin B
1.1 mM Mg^{2+} (± 0.02)	0.31	0.33
10 mM Mg^{2+} (± 0.01)	0.53	0.48

Supplementary Table 3. Thermodynamic parameters obtained by ITC titration of c-di-GMP in the absence or presence of kanamycin B.

		**2 mM Mg^{2+}**		**10 mM Mg^{2+}**	
	kanamycin B:	none	200 μM	none	200 μM
K_D	(μM)	0.978	1.350	0.270	0.378
ΔH	(kcal/mol)	-12.51	-14.03	-9.79	-10.42
$-T\Delta S$	(kcal/mol)	4.45	6.16	0.98	1.81
ΔG	(kcal/mol)	-8.06	-7.87	-8.81	-8.62

Supplementary Notes

Supplementary Note 1. Analysis of kanamycin B titrations. The biphasic kanamycin B-induced E_{FRET} responses precluded analysis using a simple two-state model. Given the unusual shape of the conformational landscape, we did not extrapolate end-points assuming monotonic E_{FRET} increases or decreases (at higher kanamycin B concentrations). Indeed, at higher kanamycin B concentrations additional non-monotonic E_{FRET} responses may exist. We therefore did not fit the ten kanamycin B titrations to any binding model.

Supplementary Note 2. Advantages of FRET thermal melts over traditional DSF experiments. Differential scanning fluorimetry, or DSF, experiments characterize ligand-conferred protein stability[4,5]. Given its easy adaptation to high-throughput instrumentation, the assay has been adopted for chemical screening against protein targets; active compounds induce a shift in thermal unfolding (T_{m}). The fluorescence signal is generated from an exogenous ligand whose emission is significantly increased upon binding hydrophobic patches exposed during protein unfolding. Potential drawbacks to traditional DSF experiments arise from the non-specific protein-dye interaction. First, an optimization step is required to identify the combination of protein and dye concentrations yielding robust signal changes. Second, some protein-dye pairs interact only weakly, resulting in small or indiscernible signal changes in the melting curve. In such cases, an alternative dye must be employed. Third, due to the random association of the dye with the protein, the thermally-induced transition cannot be structurally interpreted.

To avoid these drawbacks of traditional DSF, we developed a differential scanning FRET assay with two rationally positioned fluorescent dyes that are covalently attached to the RNA. Based on prior knowledge of the 3D structure and folding of the RNA, the location of the dyes ensures a FRET signal concomitant with folding. Furthermore, the fluorescence changes during thermal melting are not dependent on an interaction between the dye and the RNA. The dyes fluoresce under all temperatures probed and their signal change reports on the conformational change in the RNA.

Supplementary Note 3. $T_m^{3°}$, a tertiary thermal unfolding parameter. We reported the $T_m^{3°}$, or tertiary unfolding temperature, from each thermal unfolding curve. We used the $T_m^{3°}$ notation rather than the more conventional T_m notation for two reasons. First, our assay monitors tertiary RNA unfolding and needs to be distinguished from the more common qPCR application of measuring DNA duplex melting for genotyping single-nucleotide polymorphisms. Second, our FRET construct and experimental temperature range (<70 °C) only evaluated the tertiary melting transition; the duplex melting transition occurs around ~76 ºC (**Supplementary Fig. 9d**).

Supplementary Note 4. Error in the $T_m^{3°}$ measurement. The $T_m^{3°}$ landscape is generated by calculating the derivative of the E_{FRET} signals over 194 temperatures for each of the 100 multiplexed solution conditions. The high-frequency noise in the E_{FRET} measurements (**Fig. 4** and **Supplementary Fig. 6**) is inconsequential when the overall E_{FRET} signal change is large. However, when the overall E_{FRET} signal change is very small (*e.g.* the lowest Mg^{2+} concentrations in presence of c-di-GMP), this high-frequency noise becomes significant and leads to increased error in the $T_m^{3°}$ derivative calculation. The lower signal-to-noise ratio for $T_m^{3°}$ measurements at low Mg^{2+} concentrations results in minor differences in the shape of the c-di-GMP thermal stability landscape relative to the shape of the c-di-GMP

conformation landscape. These differences are not physically meaningful, but demonstrate the limitations of $T_m^{3°}$ measurements when the ΔE_{FRET} signal is small.

Supplementary Note 5. Coupling and uncoupling of RNA conformation and thermal stability. The thermodynamic hypothesis of macromolecular (protein and RNA) folding[6] asserts that the native state represents a global minimum of free energy. Hence, folded conformations and their free energy are directly linked. Therefore, the conformation and stability of productive folding intermediate states are necessarily intermediate between those of the unfolded and folded states. In contrast, non-productive trapped intermediates do not simply transition along the pathway between unfolded and folded states. They may be topologically constrained[7] or may be stabilized by non-native contacts with a protein or small molecule ligand[8]. In either case, the thermal stability of those intermediates is not inherently coupled to those of the natively folded and unfolded states. This uncoupling may result in disparate measurements of conformation, stability, or specific activity.

In our study, the incongruent E_{FRET} and $T_m^{3°}$ landscapes observed for the riboswitch-kanamycin B interaction demonstrate that the folding and stability have been uncoupled. We interpret this to be caused by a non-productive intermediate stabilized by kanamycin B. In contrast, coincident conformational and thermal stability landscapes were observed in the presence of cyclic di-GMP. This coupling between folding and stability is consistent with the absence of off-pathway intermediates.

Supplementary Note 6. Non-cognate ligand binding is highly dependent on Mg^{2+} concentration. The conformational and stability landscapes reveal that kanamycin B binding is precluded at high Mg^{2+}

conditions (25 mM). Stated otherwise, the Mg^{2+}-stabilized state is not disrupted by up to 200 μM kanamycin B. High Mg^{2+} conditions screen the affect even of non-specific charge interactions (kanamycin B has five positively charged amino groups at neutral pH) such that the $T_m^{3°}$ is nearly unchanged by the addition of kanamycin B in 25 mM Mg^{2+} (**Supplementary Fig. 10**). In contrast, the riboswitch-kanamycin B interaction observed under low Mg^{2+} conditions, where c-di-GMP does not bind, results at least in part from the positive charge of the aminoglycoside. The strong Mg^{2+} dependence of the RNA-kanamycin B interaction highlights the importance of choosing appropriate solution conditions when screening for novel ligand-RNA interactions. Non-cognate ligands are likely to bind metastable conformations, whose structures are not accessible under highly stabilizing conditions.

Supplementary Note 7. Obtaining conformational and stability landscapes from a single instrument read. To generate conformational landscapes we measured fluorescence intensities with minimal noise reliably down to 15 nM RNA (20 μL) using a microplate fluorometer. Fluorescence measurements using a qPCR machine under similar conditions yielded significantly noisier E_{FRET} measurements. However, using 40 nM RNA in 25 μL we recorded high quality E_{FRET} measurements on the qPCR machine for Mg^{2+} titrations in the absence and presence of c-di-GMP. The quality of these data produced fits to the two Mg^{2+} titrations in agreement with data obtained from the microplate fluorometer. Preparation of samples in this way will allow for robust measurement of all four parameters (E_{FRET}, K_{Mg}, K_D, and $T_m^{3°}$) and generation of conformational and stability landscapes in a single experiment using a single read on a qPCR machine.

Supplementary References

1. Wood, S., Ferré-D'Amaré, A. R. & Rueda, D. Allosteric tertiary interactions preorganize the c-di-GMP riboswitch and accelerate ligand binding. *ACS Chem Biol* **7**, 920-927 (2012).
2. Smith, K. D., Lipchock, S. V., Livingston, A. L., Shanahan, C. A. & Strobel, S. A. Structural and biochemical determinants of ligand binding by the c-di-GMP riboswitch. *Biochemistry* **49**, 7351-7359 (2010).
3. Kulshina, N., Baird, N. J. & Ferré-D'Amaré, A. R. Recognition of the bacterial second messenger cyclic diguanylate by its cognate riboswitch. *Nat Struct Mol Biol* **16**, 1212-1217 (2009).
4. Niesen, F. H., Berglund, H. & Vedadi, M. The use of differential scanning fluorimetry to detect ligand interactions that promote protein stability. *Nat Protoc* **2**, 2212-2221 (2007).
5. Pantoliano, M. W. *et al.* High-density miniaturized thermal shift assays as a general strategy for drug discovery. *J Biomol Screen* **6**, 429-440 (2001).
6. Epstein, C. J., Goldberger, R. F. & Anfinsen, C. B. The Genetic Control of Tertiary Protein Structures: Studies with Model Systems. *Cold Spring Harbor Symp Quant Biol* **28**, 439-449 (1963).
7. Chen, C. *et al.* Understanding the role of three-dimensional topology in determining the folding intermediates of group I introns. *Biophys J* **104**, 1326-1337 (2013).
8. Grohman, J. K. *et al.* An immature retroviral RNA genome resembles a kinetically trapped intermediate state. *J Virol* **88**, 6061-6068 (2014).
